# Design of prodrugs with reactive oxygen species as activators and their application in tumor therapy

**DOI:** 10.7150/thno.121977

**Published:** 2026-01-01

**Authors:** Jiaqi Xing, Wenjuan Lu, Yubing Zhang, Chen Yang, Jikai Yang, Jing Shi, Yanfeng Wang

**Affiliations:** 1 State Key Laboratory of Advanced Drug Delivery and Release Systems, Key Laboratory for Biotechnology Drugs of National Health Commission, Key Laboratory of Rare and Rare Diseases in Shandong Province, School of Pharmacy (Institute of Pharmacy) of Shandong First Medical University, Jinan, Shandong 250117, China.; 2 Department of Pharmacy (Shandong Key Traditional Chinese Medical Discipline of Clinical Chinese Pharmacy), Shandong Cancer Hospital and Institute, Shandong First Medical University and Shandong Academy of Medical Sciences, Jinan 250117, China.

**Keywords:** ROS, prodrugs, nanoparticles, tumor treatment

## Abstract

Major challenges lie in the precise management (encompassing diagnosis and treatment) of malignant neoplasms. Traditional chemotherapy faces restrictions in clinical use because of its ineffective targeting and significant toxicity, along with side effects. Notably, the ROS levels are observably elevated in cancer cells compared to healthy tissues, which presents a distinct opportunity for the creation of prodrugs that respond to ROS. This article systematically reviewed the research progress on ROS-responsive small molecule prodrugs and nanodelivery systems (including polymer/inorganic nanoparticles and hydrogels) over the past five years and elaborated in detail on the design principles based on seven key activation mechanisms. By combining ROS responsiveness with TME specificity, these systems have achieved precise controlled drug release, significantly reduced toxic and side effects, and demonstrated multiple synergistic effects of chemotherapy, immunotherapy, and photodynamic therapy. Additionally, some systems integrate theranostic and imaging functions, allowing real-time observation of the drug release. Subsequently, the latest progress in the field from molecular design to preclinical research was summarized, and the promise of ROS-responsive systems for clinical applications was emphasized. It directs the creation of prodrugs that are highly specific and supports the advancement of multi-responsive theranostic platforms, thereby paving the way for improved precision in the diagnosis and treatment of tumors.

## 1. Introduction

A crucial obstacle in global public health is the growing importance of early detection and management of malignant tumors 1. By the year 2025, it is forecasted that there will be 2 million newly diagnosed cases of cancer, with cancer-related deaths reaching 600,000. In 2022, the survival rate increased by 34% compared to 1991, but it still adds up to a huge burden. Currently, traditional methods for treating cancer mainly consist of surgery, chemotherapy, radiotherapy, and immunotherapy; however, each of these treatments has notable limitations 2,3. Due to the lack of accurate identification of tumor boundaries, surgical resection often faces a dilemma 4: incomplete resection or excessive resection. The former may lead to tumor metastasis or recurrence, while the latter tends to impair the normal physiological function of tissues and organs. Although systemic chemotherapy and radiotherapy have broad-spectrum antitumor effects, their mechanisms of cytotoxicity, which are not selective, may lead to harm to healthy tissues and organs 5. Immunotherapy offers specific targeting and minimal toxic side effects, but the high cost makes it challenging to popularize on a large scale 6. It is worth noting that in 2024, there were 2,162 oncology trials globally, coming in at 41% of all clinical trials. Moreover, solid tumors remain a key focus of research and development, reflecting the pressing demand within tumor therapy. Stimuli-responsive prodrugs and drug delivery systems (DDS) open new paths for tumor treatment. By precisely regulating drug activity and targeted delivery, these systems significantly enhance therapeutic efficacy and reduce systemic toxicity 7,8. Its activation methods are rich and diverse, such as the widely applied exogenous stimulus regulation strategy. The Perylene Diimide-Based Photoacid Generator (PBI-PAG) can respond to green light/red light (560-605 nm), efficiently release acid and photosensitizers, and achieve deep tissue penetration by utilizing the long wavelength. The synergistic effect of the released acid and photosensitizers enhance the antitumor effect 9. Camptothecin prodrug NO-CPT can be activated by the hydrated electrons generated by radiotherapy, achieving the synergistic effect of radiotherapy and chemotherapy 10. The palladium-nanomodified microneedle patch delivers prodrugs to the tumor location through biorthogonal reactions catalyzed by transition metals, featuring high targeting efficiency and minimal damage to normal tissues 11. The specificity of the tumor microenvironment (TME) as a natural trigger signal is also widely applied 12, which can reduce off-target activation by leveraging the difference between the TME and normal tissues, including reactive oxygen species (ROS), reactive nitrogen species (RNS), levels of thiols, viscosity, pH, and polarity 13. Previous reports have shown that cabazitaxel (CTX) and chitosan (CS) are conjugated through a glutathione (GSH)-sensitive disulfide maleimide (DTM) for treating breast cancer 14. In addition, acid-responsive hydrazone bonds are employed to prepare targeted nanomedicines, PEG-Dendron-EPI@TPP-LND, which exerted a synergistic effect by inhibiting oxidative phosphorylation (OXPHOS) and enhancing the chemotherapy efficacy of the epirubicin (EPI) prodrug, leading to enhanced treatment outcomes for triple-negative breast cancer 15.

ROS are essential in biochemical reactions and sustaining redox homeostasis inside the cell 16. Appropriate ROS levels are crucial for maintaining immune responses, regulating cellular signaling, and ensuring cellular homeostasis 17,18. In various diseases, there is often an abnormal increase in ROS levels 19, which is not only an essential indicator of the pathological characteristics of diseases but also provides key targets for elucidating disease mechanisms and formulating treatment strategies 20,21. Disease treatment targeting ROS has become a research hotspot. For instance, using the pathologically elevated ROS levels at the infection site as a “biological switch”, a supramolecular self-assembly system regulated by ROS has been constructed to achieve the inhibition of bacterial infection 22. The combination of ROS-induced supramolecular assembly with biorthogonal reactions enables the spatiotemporally controlled release of the inhibitory neurotransmitter GABA, thereby inhibiting epileptic seizures 23. Elevated ROS levels can also serve as a potential target for enhancing the precision of tumor diagnosis and treatment 24. The reasons for the increase in ROS levels in tumors can be divided into the following aspects: 1. Abnormal metabolism: The energy requirements rise due to heightened metabolic activity in tumor cells, which promotes mitochondrial production capacity, breaks the balance of adenosine triphosphate (ATP) metabolism in the cell, and leads to a decrease in the efficiency of oxidative phosphorylation (a key process for efficient ATP production) 18,25,26. Electron transfer in the electron transfer chain (ETC) is blocked, and it is easier to react with oxygen to generate ROS, which directly leads to an increase in intracellular ROS levels 27. 2. TME factors: The TME is often in a state of hypoxia 28,29. This triggers the activation of hypoxia-inducible factor-1 (HIF-1), which enhances the production of ROS. At the same time, the new ROS will upregulate the expression of HIF-1, establishing a positive feedback mechanism 30. Furthermore, immune cells, including macrophages and T lymphocytes, that gather around tumor cells secrete inflammatory factors and cytokines. These substances stimulate tumor cells, causing an increase in ROS production 31. 3. Activation of carcinogenic signals: In the development of tumors, a variety of key carcinogenic-related proteins and downstream signaling pathways are abnormally activated, which can induce excessive ROS. For example, the continuous activation of the nuclear factor-κB (NF-κB) signaling pathway, linked to inflammation and cell survival, promotes ROS overproduction. The nuclear factor E2-related factor-2 (NRF2) acts as a crucial regulator of the antioxidant defense system, and its abnormal activation indirectly leads to ROS accumulation through redox regulatory imbalance. Furthermore, heightened activation of the phosphatidylinositol 3-kinase (PI3K) pathway is correlated with cellular proliferation and metabolic reprogramming, stimulating ROS production by changing the metabolic state of cells 32,33. Intelligent DDS that utilize the excessive production of ROS in tumor cells as specific trigger signals have attracted extensive attention 34-36. Currently, the ROS-responsive prodrug systems mainly include polymer-based nanocarriers 37-40, hydrogels 41,42, inorganic nanoparticle-based systems 43, and ROS-activatable prodrugs 44,45. By coupling drug release with the ROS levels in tumor cells, these systems achieve precise therapy for tumor cells, providing a new strategy for tumor therapy 46.

This article reviewed the latest strategies of ROS-responsive anti-tumor DDS, which mainly include the following four aspects: 1. Drug-fluorophore conjugated small-molecule prodrugs with drug-fluorophore: small molecule prodrugs obtained by chemically modifying small molecule drugs are activated by ROS in tumor cells and release active parent drugs 47. Additionally, when ROS activates the prodrug, it not only releases the active drug but also induces a change in fluorescence signal, thus enabling real-time monitoring of drug release 48. This modification method has become widely accepted to improve the chemical and metabolic stability of drugs, as well as their water solubility or lipophilicity, extend the duration of drug action, and mitigate adverse reactions. 2. ROS-responsive polymer nanoparticles (PNPs): PNPs can be synthesized using either natural or synthetic materials, resulting in diverse structures and properties 49. Drugs can be loaded into PNP-based DDS via encapsulation within the PNP core, embedding in a polymer matrix, chemical coupling with the polymer, or adsorption onto the PNP surface 50. These PNPs can carry hydrophobic and hydrophilic compounds, including small molecules 51, biomacromolecules, proteins 52, and vaccines 53. After systemic administration, PNPs preferentially concentrate at tumor locations due to the enhanced permeation and retention (EPR) phenomenon 54. Their surface-modified target ligands enable more effective intracellular transport through receptor-mediated endocytosis. Furthermore, the ROS response unit is introduced into the polymer nanoplatform to build an intelligent DDS, which can precisely control drug release at the target in a spatiotemporal controllable manner 55. 3. Hydrogel DDS triggered by ROS: Hydrogels consist of a three-dimensional network that results from the straightforward reaction of one or several monomers and contain a lot of water. The drug release mechanism is regulated by mesh size 56. When the drug molecule is larger than the mesh size, it is physically encapsulated within the mesh. With the degradation of the hydrogel network, the mesh size increases, allowing the drug to diffuse freely 57. Stimuli-responsive groups have been widely used in the development of smart hydrogels 58. Various ROS-responsive units can induce oxidative features in polymer chains or alter their hydrophobicity to hydrophilicity through bond degradation, thereby altering the mesh size and controlling drug release 59,60. 4. ROS-responsive inorganic nanoparticles: Inorganic nanoparticles, including gold, iron, and silica, have been utilized to create nanostructured materials, finding extensive application in drug delivery and imaging 61. For example, free radicals on the surface of mesoporous silica (SiO₂) nanomaterials may trigger the formation of ROS through hydration reactions. In contrast, mesoporous titanium dioxide (TiO₂) has been demonstrated to be a highly effective agent for photodynamic therapy (PDT) 62. Therefore, SiO_2_ and TiO_2_ have significant advantages in constructing ROS-responsive DDS. By grafting a ROS-responsive polymer onto the surface of mesoporous-structured inorganic materials, using the inorganic material as a carrier, and combining the stimulus response of the polymer, the functions of drug encapsulation and controlled release can be realized 63. In summary, this review systematically summarized the design strategy of ROS-activated anti-tumor drug delivery platforms, demonstrating their great potential for tumor diagnosis and treatment.

**Figure 1:**
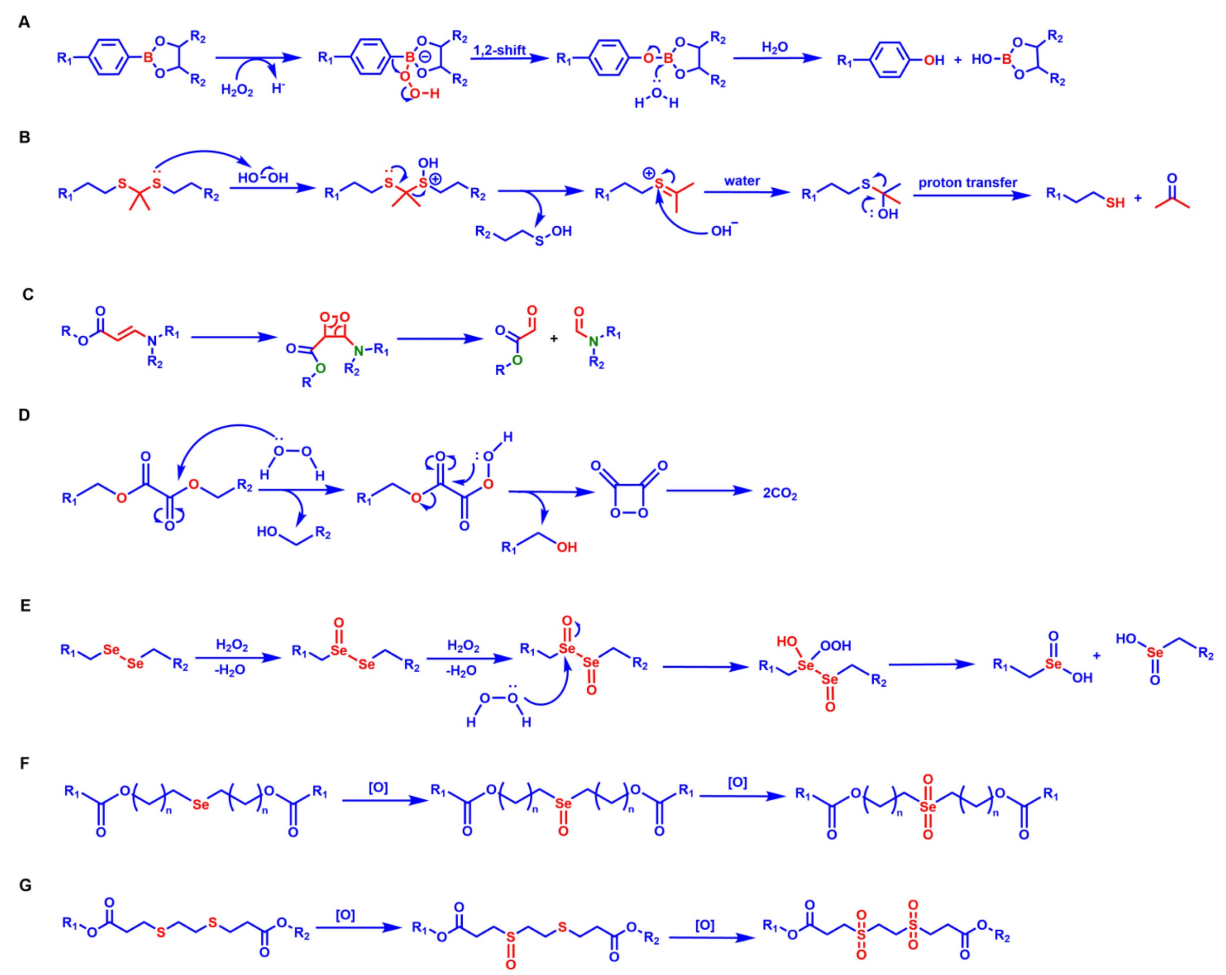
The response mechanisms of different ROS-responsive groups. (A) Phenylborate ester reacts with H_2_O_2_ under acidic conditions and undergoes hydrolysis after a 1,2-shift, generating phenol and hydroxyborate ester. (B) Thioketal undergoes oxidation, hydrolysis, and proton transfer, producing thiol and carbonyl compounds. (C) Aminoacrylate is oxidized and cleaved via a peroxide-ring intermediate, forming carboxylate ester and imide. (D) The peroxalate ester undergoes oxidation-induced rearrangement and the formation of cyclic peroxide, ultimately resulting in the release of CO_2_. (E) Diselenide linkers are stepwise oxidized by H_2_O_2_, yielding selenium-containing oxidation products. (F) Selenium ether undergoes a two-step oxidation, first forming a selenoxide and then a selenone. (G) The thioether undergoes a two-stage oxidation, initially generating a sulfoxide and then a sulfone.

The response site in ROS-sensitive prodrugs is currently divided into two categories: cleavable linking groups and non-cleavable linking groups 44. The cleavable linking groups mainly include boronic acid and boronate (e.g., phenylboronic acid and phenylboronate) 64, thioketal 65, diselenide 66, peroxalate ester 67, vinyl disulfide, polysaccharide, and aminoacylate, among others. The non-cleavable linking groups include the chalcogen ether family (thioether, selenium ether, tellurium ether) (Figure 1). Hydrogen peroxide (H_2_O_2_) can oxidize the C-B bond in boronic acid 68. In this reaction, a nucleophile engages with the boron atom, resulting in the creation of a boron anion. Following this, the phenyl group moves to the initial oxygen atom via a 1,2-shift, leading to the displacement of the hydroxyl group. The oxidation intermediate then undergoes hydrolysis with water, generating phenol and boric acid derivatives. Upon exposure to H_2_O_2_, the lone pair of electrons on the sulfur atom within the thioketal engages with the oxygen atom of H_2_O_2_, which facilitates the creation of a sulfur cation. Simultaneously, the lone pair of electrons from the nearby sulfur atom donates electrons to the adjacent carbon, causing the thioketal backbone to break and resulting in the liberation of sulfenic acid 68. The leftover residue undergoes additional hydrolysis, eventually producing thiol and acetone, with the destruction of the thioketal structure. The initial stage of aminoacrylate's response to ROS involves singlet oxygen (¹O₂) directly oxidizing double bonds to form a four-membered ring structure. Subsequently, the ring collapses, producing two aldehyde groups and releasing the payload 69. H_2_O_2_ acts as a nucleophile to attack the peroxalate ester and directly remove alcohol groups with R_2_ groups. Subsequently, another oxygen atom undergoes a similar reaction, resulting in the liberation of the R_1_ group. This sequence of reactions eventually forms 1,2-dioxetanediones and four-membered ring intermediates, which are then converted into carbon dioxide 70. The cleavage process of diselenide linkers begins with two molecules of H_2_O_2_ successively oxidizing selenium to selenium oxide. Subsequently, H_2_O_2_ can attack selenium atoms, leading to the creation of intermediate compounds containing hydroxyl and peroxide functional groups. This intermediate subsequently breaks down into two selenious acids by disrupting the Se-Se bond 71. The oxidation process of selenium ether is similar to that of thioether, involving the gradual addition of oxygen to sulfur/selenium atoms, with the molecular polarity continuously increasing until it reaches its maximum value, thereby completing the transformation from hydrophobicity to hydrophilicity without breaking the chemical bond.

There is significant heterogeneity in ROS levels among different tumor tissues, and it can serve as the basis for selecting ROS-responsive groups 72. Tumors with high ROS levels, such as glioblastoma (GBM) and esophageal squamous cell carcinoma (ESCC), can preferentially use boric acid and its ester, selenide, thioether, and thioketal responsive groups. Among them, thioketals have adjustable ROS sensitivity, and the oxidative degradation rate of thioketals can be controlled by changing the substituents 73,74. The natural activation threshold of thioethers is relatively high, so it is necessary to use them in combination with ROS amplifiers. Boric esters exhibit unique advantages for tumors with moderate ROS levels, such as pancreatic cancer (PAAD) and some lung adenocarcinomas (LUAD). In tumor environments that are mildly acidic (pH 6.0-6.8), boric esters can work synergistically with H₂O₂ to accelerate the hydrolysis efficiency, and the drug release rate is 5-10 times greater compared to neutral conditions (pH 7.4) 75. For tumors with low ROS levels, such as adrenal cortical carcinoma (ACC), diselenide linkers can be selected to achieve sustained drug release and maintain long-term therapeutic concentrations. In addition, the aminoacrylate bond has a specific response to ¹O₂ and is usually used in combination with photosensitizers under laser irradiation. The large amount of ¹O₂ produced by the photosensitizer can effectively trigger cleavage of the aminoacrylate. This “light-controlled ROS response” mode is particularly suitable for the treatment of superficial tumors 76.

## 2. ROS-Activable Small Molecule Prodrugs

In the field of organic synthesis, the protection of active functional groups followed by deprotection is a common approach used to prevent adverse side effects 88. This synthetic strategy has been applied to drug development, leading to the conception of prodrugs. Prodrug design involves conjugating specific protective groups to the active moiety of a known drug 89. In non-healthy cells, these protective groups can be removed by enzymes or molecules, thereby restoring the pharmacological activity of the parent drug 90. Prodrug strategies can enhance the targeting selectivity and efficacy of drugs, mitigate side effects resulting from insufficient drug solubility or low cellular uptake, and ultimately improve the overall pharmaceutical properties of drugs 91.

**Figure 2:**
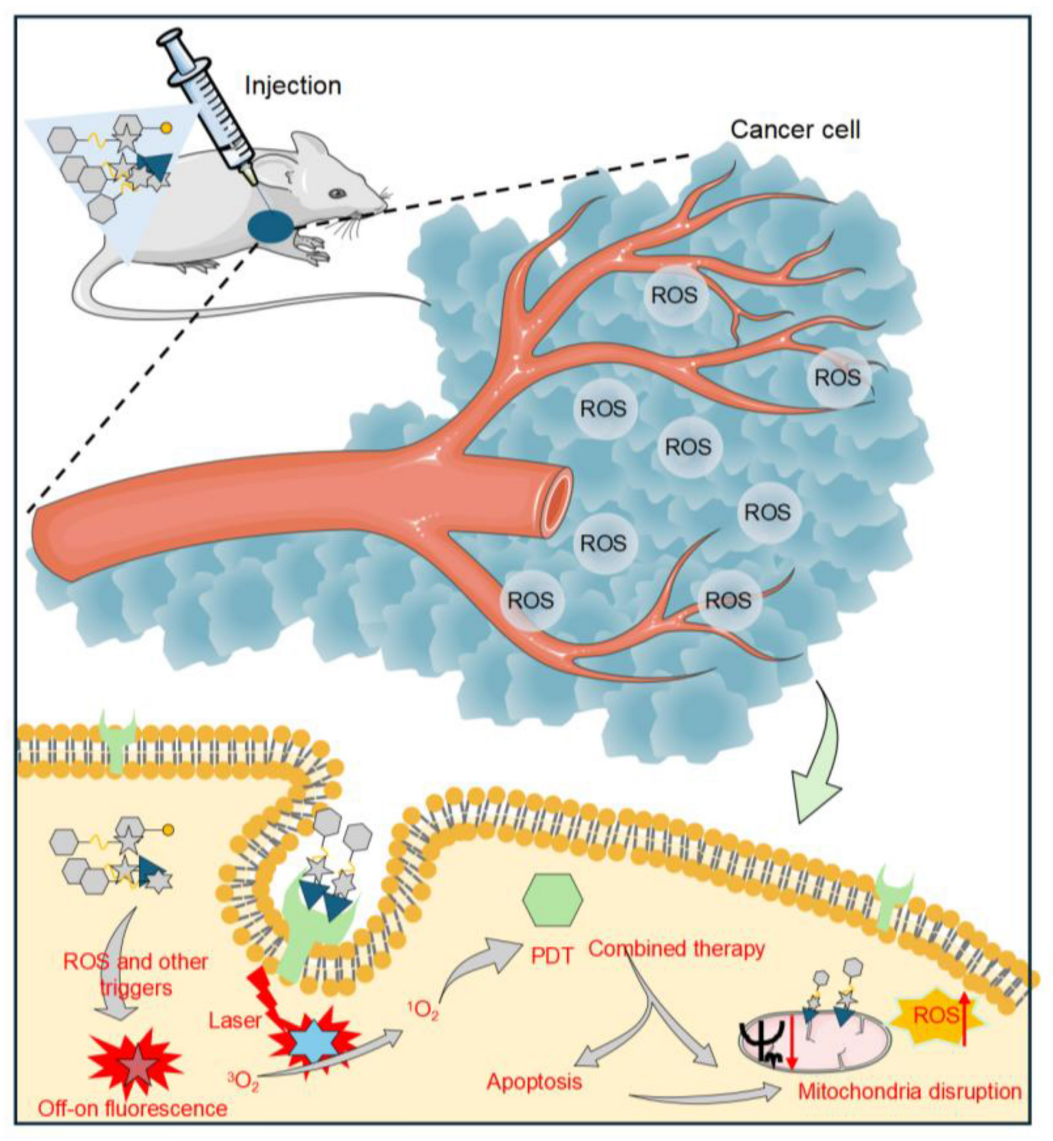
A schematic representation of how small-molecule prodrugs function in the treatment of tumors. After administration, the prodrug is transported to the tumor location and activated within the TME, which is marked by elevated levels of ROS. The fluorophores integrated into the prodrug exhibit an “on-off” fluorescence response during activation and functional depletion, facilitating the real-time monitoring of prodrug activation and its therapeutic effects. Under laser irradiation, the photosensitizer portion within the prodrug generates ¹O₂. ¹O₂ works synergistically with chemotherapeutic drugs released by activated prodrugs, destroying mitochondrial function and ultimately inducing apoptosis of cancer cells. Meanwhile, targeting groups in the prodrug allow for specific recognition and binding to highly expressed receptors on tumor cells, enhancing the delivery accuracy and therapeutic specificity.

Small-molecule prodrugs offer several advantages, including low molecular weight, high drug loading efficiency, well-defined chemical structures, ease of monitoring their metabolic process, and feasibility of safety evaluation 92. Their design strategy mainly involves linking the active pharmaceutical ingredient to a specific protective group (promoiety). After administration, the drug-promoiety linkage is cleaved via chemical or enzymatic reactions to activate the parent drug. In comparison to normal cells, levels of ROS in cancer cells are approximately ten times higher 93. This disparity in concentration underpins the development of targeted prodrugs that respond to ROS. In recent years, various studies have shown that ROS can effectively serve as a trigger signal for activating prodrugs in cancer treatment, successfully applying this strategy to design ROS-responsive small-molecule prodrugs, which enable targeted drug delivery and precision cancer therapy 94 (Figure 2).

Boronic acids and their esters are commonly used as H_2_O_2_-responsive groups; carbon-boron bonds are cleaved via H_2_O_2_ oxidation, making them ideal protective moieties for the active functional groups of small-molecule prodrugs 95. Doxorubicin (DOX) is an effective antitumor drugs in the clinic; however, its clinical application is limited due to dose-dependent cardiotoxicity caused by excessive H_2_O_2_ when it exerts its effect in *vivo* 96.

**Figure 3:**
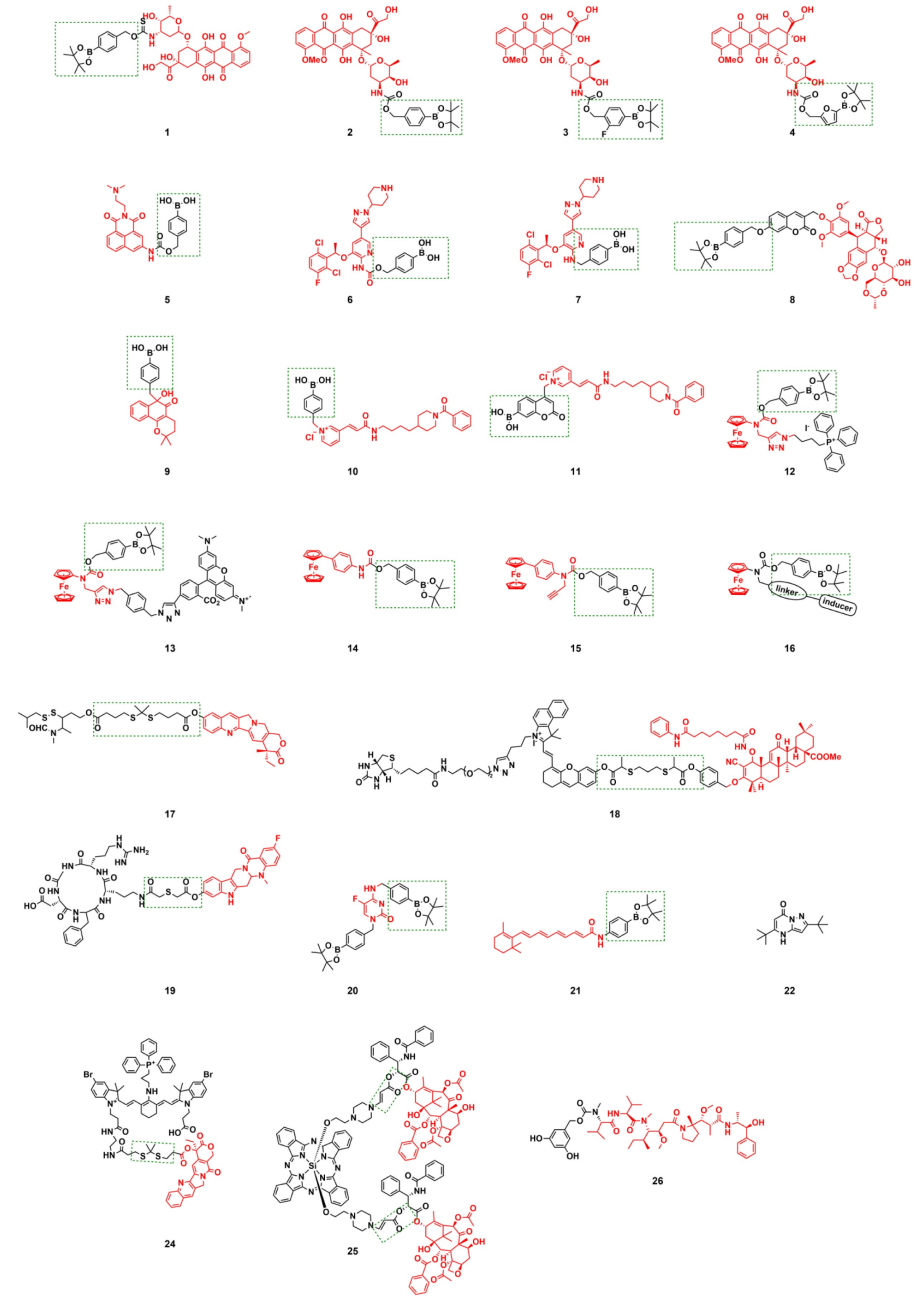
The chemical architecture of prodrugs that respond to ROS. The prodrug depicted in the figure enables site-specific activation and drug release in ROS-enriched TME by coupling the chemotherapy drug (highlighted in red) with a ROS-reactive group (in the green box).

H_2_S is a cardioprotective agent with anticancer activity 97. Lukesh III *et al.* 98 synthesized prodrug **1** (Figure 3) by connecting boronate with DOX via the structure of carbonyl sulfide. H_2_O_2_-activated prodrug **1** within the TME and simultaneously released DOX along with H_2_S. H_2_S directly scavenged ROS, enhanced the endogenous antioxidant system, and alleviated DOX-induced cardiotoxicity. Compared with DOX, prodrug **1** reduced apoptotic effects on cardiomyocytes by releasing H_2_S, and the dose of prodrug **1** did not impede Nrf2 activation or cardiomyocytes' HO-1 expression, thus minimizing cytotoxicity. Therefore, prodrug **1** had significant potential as an alternative to DOX for reducing cardiotoxicity while retaining antitumor efficacy.

The effect of the prodrug formed by combining DOX with arylboronic acids on different cell lines varies significantly, which affects its potential for entry into clinical trials. Therefore, it is crucial to identify tumor cell lines sensitive to varying subtypes of arylboronic acid prodrugs. Labruère *et al.* 99 synthesized a series of H_2_O_2_-sensitive prodrugs, including non-substituted boronate prodrug **2**, fluorinated phenol analogs prodrug **3**, and furan-substituted boronate prodrug **4** (Figure 3). They evaluated the H₂O₂-induced activation efficiency and DOX release efficiency in different tumor cell lines. The results showed that prodrug **2** exhibited the best anti-tumor activity and the highest selective activity against pancreatic cancer cells, with the recovery rate of the active drug reaching 67% of the activity of free DOX. The effect of prodrug **2** was comparable to that of similar free DOX in the MiaPaca-2 tumor model. Yin *et al.* 100 developed an H_2_O_2_-responsive theranostic prodrug **5** based on amonafide (AMF) (Figure 3), which could be used to compare and quantify the H_2_O_2_ levels of different cells. Experimental results confirmed that the intracellular H_2_O_2_ concentration was positively correlated with the anticancer activity of prodrug **5**.

Crizotinib is a tyrosine kinase inhibitor. The 2-aminopyridine functional group in its structure can interact with the amino acids in the ATP-binding sites of the three targeted kinases (Anaplastic Lymphoma Kinase, cellular Mesenchymal-epithelial Transition factor, and ROS proto-oncogene 1, receptor tyrosine kinase), which is regarded as an ideal structural unit for prodrug modification 101. Kowol *et al.* 102 conjugated the 2-aminopyridinium moiety of crizotinib to phenylboric acid via a covalent linker to prepare prodrugs** 6** and **7** (Figure 3). Boronic acid as a ROS-responsive trigger fragment, and the 2-aminopyridinium moiety as the key site for target kinase binding. The study found that H_2_O_2_ activated prodrug **6** more easily, and the activity level of prodrug **6** was significantly and positively correlated with the intracellular H_2_O_2_ concentration. Therefore, the strategy of converting crizotinib into a prodrug can reduce systemic side effects while improving tissue selectivity for tumor cells.

Coumarin is commonly used as a tracking agent when the prodrug is released, especially for the prodrugs modified with boronic acid groups. Yu *et al*. 103 synthesized peroxide-sensitive prodrug **8** (Figure 3) by conjugating etoposide to phenylboronate via a coumarin linker. Etoposide is a topoisomerase II inhibitor that induces the death of tumor cells by creating complexes involving topoisomerase II and DNA. Although etoposide is widely utilized as a first-line chemotherapy, its use in clinical settings is hampered by cardiac toxicity, hematological toxicity, and gastrointestinal toxicity. Prodrug **8** was specifically triggered in cancer cells to release etoposide, and it exhibited similar anti-tumor activity to that of etoposide, along with enhanced safety and reduced toxicity. Due to the presence of coumarin, the activation of prodrug **8** resulted in a notable rise in fluorescence intensity within tumor cells; therefore, prodrug **8** was expected to be a safe and effective anticancer chemotherapeutic agent.

**Figure 4:**
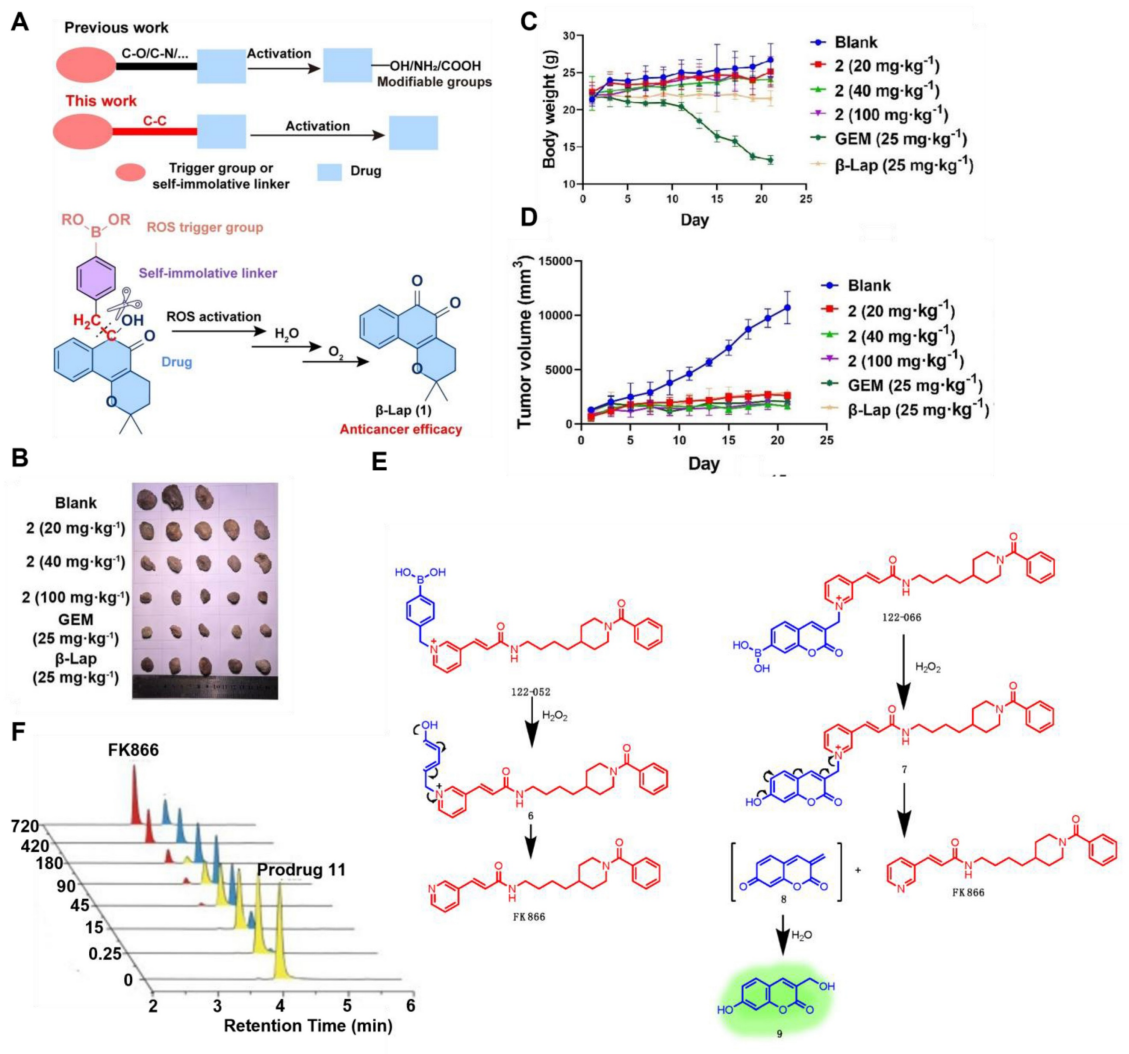
(A) A new prodrug strategy for activating ROS-reactive anticancer prodrugs through C-C bond cleavage. (B) Tumor image after compound treatment of Mia PaCa-2 xenograft in nude mice. (C) The weight of the tumor during treatment. (D) The volume of the tumor after treatment. Produced with permission 104. Copyright 2022, Wiley-VCH GmbH. (E) Diagram of the process of FK866 release from the prodrug **11**. (F) Conversion of prodrug **11** to FK866 monitored by HPLC. Reproduced under terms of the CC BY 4.0 license 105. Copyright 2020, by Jiang *et al*.

Current boronate prodrugs face substantial challenges in altering active functional groups within parent drugs. Zhang *et al.* 104 proposed a prodrug activation strategy based on C-C single bond cleavage and successfully designed a β-lapachone (β-Lap)-based prodrug **9** (Figure 3). Under the induction of ROS, the C-C bond in the prodrug structure was rapidly cleaved, releasing the parent drug β-Lap. β-Lap exerted anticancer effects through an effective redox cycle mediated by NAD(P)H: quinone redox reductase 1 (NQO1) and exhibited significant selectivity for NQO1-overexpressing tumor cells (Figure 4A-D). This method offered a new perspective on the exploitation of prodrugs from traditional drugs.

Nicotinamide phosphoribosyltransferase (NAMPT) is a rate-limiting enzyme widely present in tumor cells, has the potential to be targeted by inhibitors for cancer therapy. However, the dose-limiting toxicity of them has hindered their clinical application. Jiang *et al.* 105 reported NAMPT prodrugs **10** and **11** (Figure 3), which could be activated by ROS, and the toxicity was significantly reduced in normal cells compared to their parent NAMPT inhibitor. Moreover, because prodrug **11** contained a coumarin fluorophore, its fluorescence signal changed when the parent drug was released from it under H_2_O_2_ activation (Figure 4E-F).

**Figure 5:**
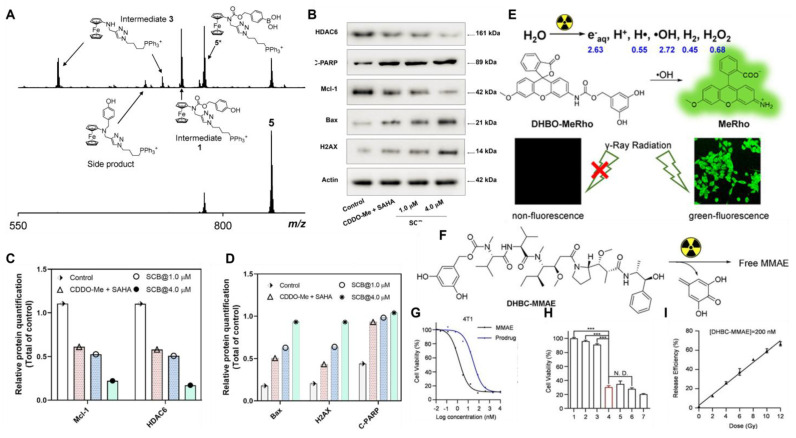
(A) Mass spectra (obtained through electrospray ionization (ESI) in positive mode) for the prodrug **12** (at a concentration of 40 μM in N,N-dimethylformamide DMF/CH_3_CN/H_2_O, in a ratio of 1/10/90, v/v/v) were recorded with H_2_O_2_ present (35 mM, top) and absent (bottom) at a temperature of 22℃. Reproduced under terms of the CC BY 4.0 license 106. Copyright 2020, by Mokhir *et al*. (B) Impacts of 1.0 μM CDDO-Me combined with 1.0 μM SAHA and SCB (1.0 and 4.0 μM), on the expression of proteins associated with apoptosis and anti-apoptosis in A549 cells. (C, D) Quantitative assessment of Mcl-1, HDAC6, Bax, H2AX, and C-PARP in various treatment conditions. Reproduced with permission 109. Copyright 2022, American Chemical Society. (E) A schematic diagram showing the selective and effective removal of DHBC as a masking group by OH• generated by external radiation. (F) Chemical structure of the MMAE prodrug. (G) The cell viability of 4T1 cells after co-incubation with MMAE and prodrug **26**. (H) MMAE in vitro controlled-release cell viability assay ([prodrug **26**]=10 nm, n=5, double-tailed unpaired Student's t-test, ***P<0.001). (I) Drugs with radiation dose gradient release ([prodrug **26**]=200 nm, Co-60 as the γ radiation source, 1 Gy/min). All samples were incubated for 2 hours before analysis. Reproduced with permission 110. Copyright 2022, Wiley-VCH GmbH.

Delivering drugs to organelles lifts the anticancer effect. Mokhir *et al.* 106 synthesized prodrugs **12** and **13** (Figure 3) with mitochondrial targeting function by coupling N-alkylamine ferrocene (NAAF) to an alkyl triphenylphosphine (TPP) carrier and N,N,N'N'-tetramethylrhodamine, respectively. Prodrugs could form p-quinone methyl, CO_2_, intermediates 1, 2, 3, and drugs in a medium containing a large amount of ROS (Figure 5A). Experiments showed that both prodrugs could accumulate and be activated directly in the mitochondria of tumor cells. The mechanism of action involved weak ROS produced by NAAF modulating mitochondrial membrane potential (a process facilitated by TPP-mediated mitochondrial targeting). This discovery opened new opportunities for the advancement of cancer research and the development of treatments. However, aminoferrocene-based drugs were unstable under oxidative conditions, which led to their easy inactivation in tumor cells. Subsequently, the team selected 4-ferrocenyl aniline (4-FcAn), which was more stable than ferrocenylamino-ferrocene (AF), for the synthesis of prodrugs **14** and **15** 107 (Figure 3). Under aerobic conditions, 4-FcAn could remain stable in buffer solution for an extended period and facilitated the production of more reactive OH• from H₂O₂ via a Fenton-like reaction. Prodrugs **14** and **15** were superior to prodrugs **12** and **13** in terms of ROS-generating capacity and antitumor efficacy. Wang *et al.* 108 designed and synthesized boronate prodrugs of NAAF, and conjugated these prodrugs with Glutathione Peroxidase 4 (GPX4) inhibitors (RSL3, ML162, and ML210) to form prodrug **16** (Figure 3). The prodrug **16** exhibited higher ferroptosis selectivity and more potent anticancer activity, thereby addressing the selectivity and toxicity issues associated with GPX4 inhibitors.

**Figure 6:**
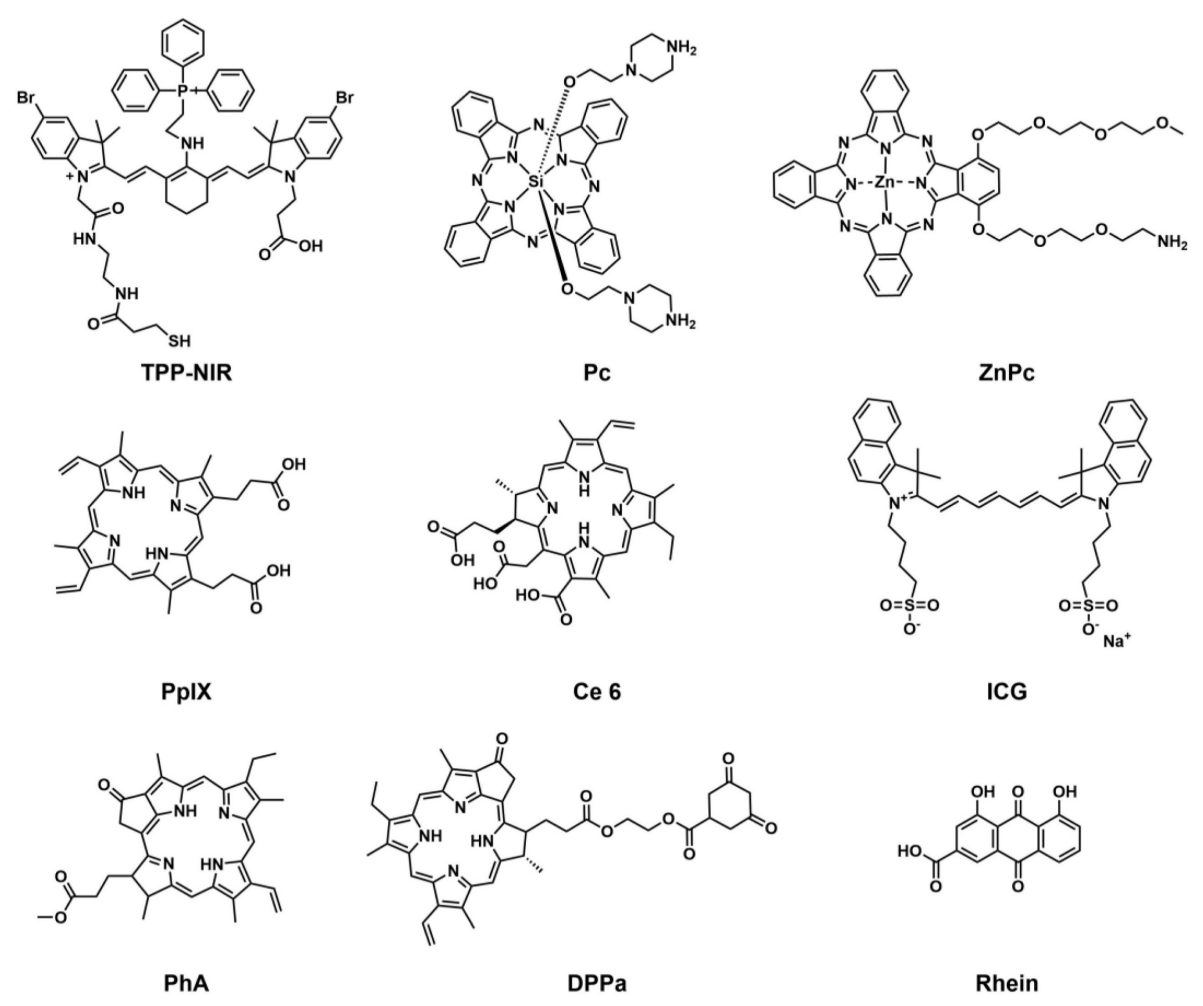
The chemical structure of photosensitizers and sonosensitizers in ROS-reactive tumor treatment systems. They can produce ROS under light or ultrasonic stimulation. Both are key components for amplifying ROS levels in the TME or triggering ROS-mediated activation of prodrugs.

Different ROS or GSH levels in different regions or stages of tumor tissues result in heterogeneous redox states, leading to partial activation of the ROS-responsive prodrug. To further improve the activation level, Wu *et al.* 111 conjugated GSH-sensitive thiamine disulfide (TDS) to 10-hydroxycamptothecin (HCPT) through a thioketal bond to obtain prodrug **17** (Figure 3). TDS reacted with GSH to generate intermediates with substantial positive charges, which could effectively remain in tumor cells and prevent efflux via charge-mediated interactions. High levels of ROS subsequently activated this intermediate, releasing HCPT. This dual-responsive design optimized the targeting ability of prodrug 17. Ling *et al.* 109 synthesized ROS/GSH dual-responsive prodrug **18** (Figure 3) using bis(sulfanediyl) dipropionate. This prodrug could attenuate the toxicity of suberoylanilide hydroxamic acid (SAHA) and C-28 methyl ester of 2-cyano-3,12-dioxoolen-1,9-dien-28-oic acid (CDDO-Me) in normal tissues, thereby reducing their related side effects and enabling a synergistic approach to combination chemotherapy (Figure 5B-D). Furthermore, prodrug **18** was equipped with a biocompatible fluorescent dye, indocyanine green (ICG) (Figure 6), and a biotin targeting moiety. This design increased the proportion and kinetic rate of prodrug activation in tumor tissues, providing a novel safety framework for accurate diagnosis and guidance for tumor resection and selective combination therapy. Hu *et al.* 112 synthesized prodrug **19** (Figure 3), which attaches the αvβ3-targeting cyclic peptide cRGD to 3-fluoro-10-hydroxy-Evodiamine (F-OH-Evo) via a thioether bond, allowing the swift release of the parent drug while exhibiting improved anti-tumor migration activity.

The prodrugs above were designed based on the higher ROS levels in the TME. Additionally, exogenous stimuli such as light, ultrasound, and heat can either activate prodrugs directly or induce ROS production in the TME, thereby facilitating ROS-mediated drug release. Wende *et al.* 113 synthesized 5-fluorocytosine prodrug **20** incorporating an aryl boronate moiety and utilized cold physical plasma (CPP) to generate ROS (Figure 3). They found that CPP triggered the ROS reaction of prodrug **20,** releasing the drug (Figure 5E-G). Using this strategy, the same team 114 also synthesized prodrug **21** (Figure 3), in which the aryl boronate group was used to mask the highly reactive hydroxyl group of fentretinoin, reducing its chemical reactivity, improving its aqueous solubility, and enhancing its selectivity toward tumor cells. CPP-induced ROS and RNS triggered the release of fentitic acid from prodrug** 21**, reducing adverse side effects. These results indicated that CPP-reactive prodrugs are valuable for further study.

Similar to CPP, cold atmospheric plasma (CAP) can regulate the source of ROS and RNS, thereby promoting drug release. Curtin* et al.* 115 synthesized pyrazolopyrimidinone prodrugs **22** and **23** (Figure 3) and combined the prodrugs with CAP to study the anti-tumor effect. The combination of **22** and **23** with low doses of CAP demonstrated cytotoxicity that was 15 and 5 times greater in U-251MG cells, respectively. The result displayed that the combination of CAP and pyrazolpyrimidinone could activate prodrugs locally in tumors, thereby minimizing the effects on other tissues and providing an innovative strategy for creating pyrazolpyrimidinone prodrugs.

Thioketal bonds can be selectively cleaved by H_2_O_2_ and exhibit strong modifiability, making them ideal connecting groups for the activation of ROS. Gao *et al.* 116 utilized thioketal bonds to link the chemotherapeutic drug camptothecin (CPT) with the photosensitizer TPP-NIR (Figure 6), introducing a mitochondrial targeting group, TPP, to obtain the ROS-activated anticancer prodrug **24** (Figure 3). After the prodrug entered the mitochondria of the tumor cells, TPP-NIR produced ^1^O_2_ under light irradiation, significantly increasing the level of mitochondrial reactive oxygen species (mtROS). High concentrations of ROS broke the thioether bond, releasing CPT and initiating mitochondrion-mediated apoptosis. Compared to previously reported ROS-activated CPT prodrugs, prodrug **24** leveraged photodynamic therapy (PDT) to achieve superior spatiotemporal control in cancer treatment, thereby minimizing damage to healthy tissue. Using the same strategy, You *et al.* 117 developed prodrug **25** by linking paclitaxel to the fluorescent photosensitizer phthalocyanine (Pc) (Figure 6) with a ^1^O_2_-cleavable aminoacrylate linker (Figure 3), which produced ^1^O_2_ under far-infrared light irradiation, triggered direct photodynamic damage, and released paclitaxel specifically at the irradiation site. In *vitro* experiments with SKOV-3 ovarian cancer cells demonstrated potent cytotoxicity (IC_50_ = 3.9 nM). This innovative approach combined PDT with site-specific paclitaxel chemotherapy, presenting a hopeful method for eliminating cancer.

In addition to extensively exploring the synergistic application of ROS-responsive prodrugs with PDT, many studies have reported their combined regimens with radiotherapy. The challenges associated with using PDT for deep tumors have been addressed due to the profound ability of radiation to penetrate deep tissues. As a masking group, 3,5-dihydroxybenzyl carbamate (DHBC) could be specifically and effectively removed by OH• generated by external radiation (Figure 5E). Liu *et al.* 110 synthesized the radiation-activating prodrug **26 (**Figure 3**)**. At the clinical standard dose of 4 Gy, the release of MMAE dramatically reduced the activity of 4T1 cells, demonstrating high in *vitro* cleavage efficiency and potential therapeutic effects (Figure 5F-I).

## 3. ROS-Activatable Polymeric Nanoprodrugs

**Figure 7:**
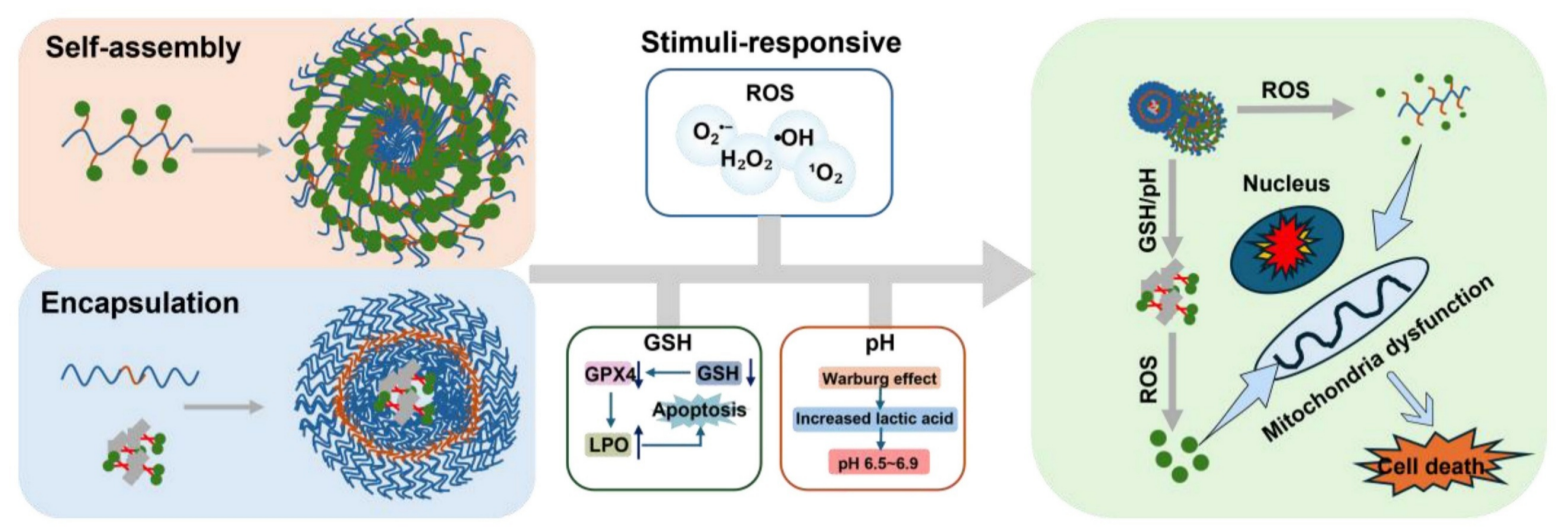
A schematic illustration depicting how ROS-reactive polymer nanomedicines function in the treatment of tumors. This figure systematically describes the multi-step process of polymer nanoprodrugs, including self-assembly, tumor targeting, time-triggered and activated release, controlled drug release, and synergistic anti-tumor treatment, to achieve targeted and efficient tumor elimination.

Small-molecule prodrugs activated by ROS show significant potential for tumor treatment because of their specific activation in the TME. However, these compounds have significant limitations: their unsatisfactory pharmacokinetic properties make it difficult to achieve targeted and precise drug delivery, and rapid metabolism in the body is also quite prominent. These deficiencies significantly limit their therapeutic efficacy and clinical application. In contrast, the physical properties (such as morphology and size) of polymeric nanoparticles can be precisely controlled by regulating the hydrophilic-lipophilic balance and optimizing the preparation process, thereby effectively improving their biological distribution in *vivo* 52. Based on the above context, scientists have successfully developed ROS-activated polymer nanomedicine systems for cancer treatment 40. After systemic administration, these systems concentrate at tumor locations and dispense medications precisely at the target site via a spatiotemporally controllable mechanism 118. By introducing targeted ligands to modify the surface of nanoprodrugs, the intracellular delivery efficiency of drugs can be further enhanced *via* receptor-mediated endocytosis 40,119. This innovative design is anticipated to address the shortcomings of conventional small-molecule prodrugs. In the past few years, notable advancements have been achieved in the creation of ROS-responsive polymer nanomedicines. This section systematically classifies these nanomedicines into four categories based on molecular design principles and response mechanisms: I. ROS-responsive groups are introduced into the polymer to construct ROS-responsive PNPs, which are then used to encapsulate therapeutic drugs. II. The polymer is linked to the drug via a ROS-responsive connector, and polymer nanoprodrugs are constructed. III. Stimulus-responsive nanomaterials can be used to encapsulate ROS-responsive small-molecule prodrugs, which are released under multiple stimuli, effectively avoiding the release of chemotherapeutic drugs in normal tissues while retaining the advantages of nanoprodrugs. Strategy IV is similar to Strategy III, except that it utilizes iron-coordinated nanocarriers to encapsulate small-molecule prodrugs, thereby constructing polymer nanocarriers (Figure 7).

### 3.1. Introducing ROS-responsive groups into polymers to construct polymer nanoprodrugs

**Figure 8:**
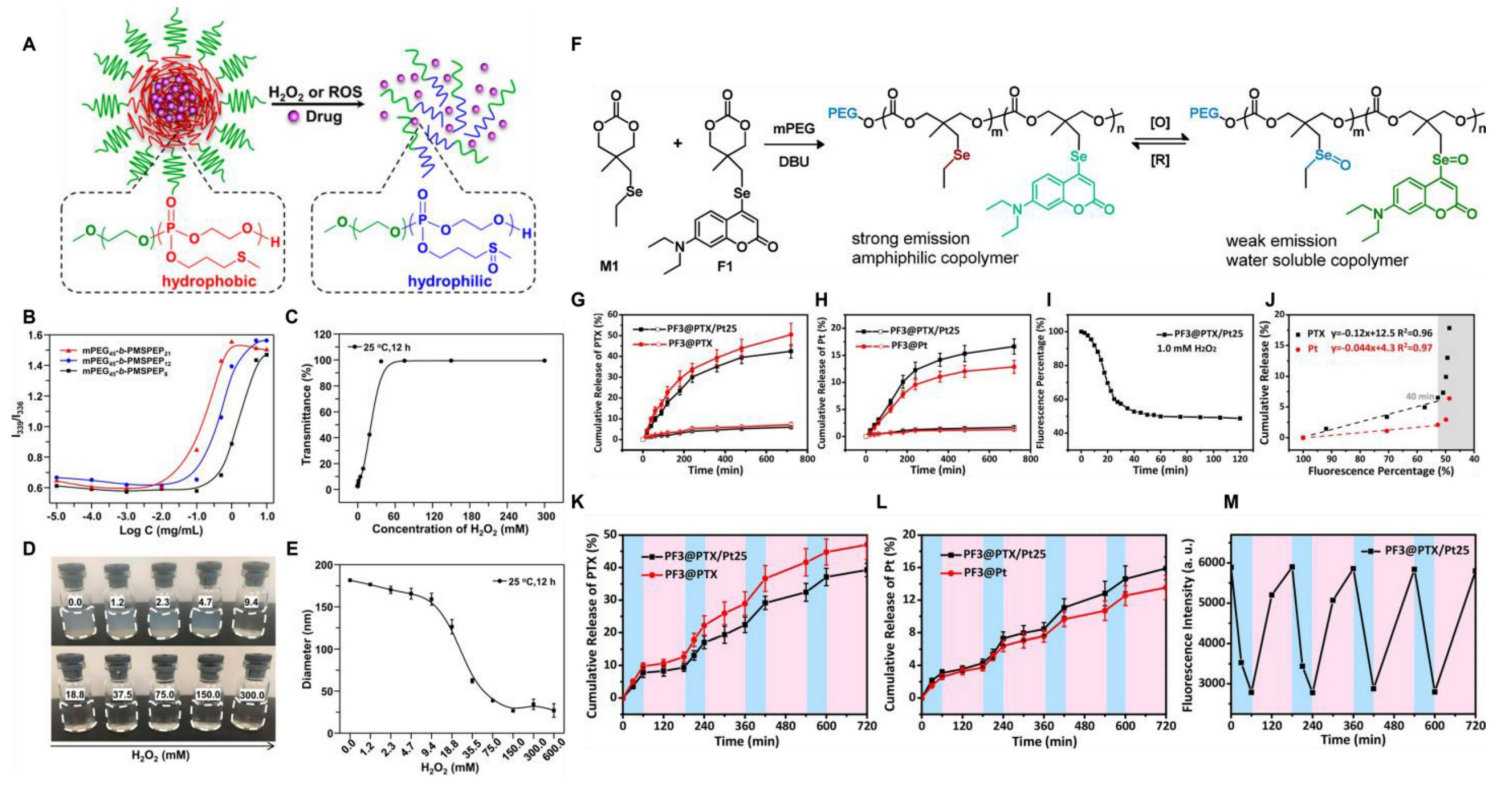
(A) mPEG-*b*-PMSPEP undergoes ROS- or H_2_O_2_-triggered cleavage to release the encapsulated drug. (B) Intensity ratio (I_339_/I_336_) of mPEG-*b*-PMSPEP at different concentrations. (C) Oxidation response behavior of mPEG_45_-*b*-PMSPEP_21_ when exposed to varying amounts of H_2_O_2_. (D) Images of nanoparticles incubated in H_2_O_2_ solutions of different concentrations. (E) Diameter changes of nanoparticles following co-incubation with varying concentrations of H_2_O_2_. Reproduced with permission 122. Copyright 2019, American Chemical Society. (F) The synthesis process of fluorescent copolymers and their reversible redox reaction transformation. (G, H) 1.0 mg/mL PF3@PTX/Pt25 nanoparticle in PBS (pH 7.4, 10 mM) with (solid symbol) or without (empty symbol) H_2_O_2_ (1.0 mM), PTX's (G) and cisplatin's (H) cumulative release at 37°C. (I) variation of the percentage of fluorescence of PF3@PTX/Pt25 over time. The data is normalized relative to the initial strength. (J) Accumulative release of drug from PF3@PTX/Pt25 plotted against fluorescence percentage. (K, L) At 37°C, the cumulative release of PTX (K) and cisplatin (L) from PF3@PTX/Pt25 (1.0 mg/mL) in PBS (pH 7.4, 10 mM) was achieved through four REDOX cycles. The results are expressed as mean ± standard deviation. (M) Variation of fluorescence intensity over time. The data is normalized relative to the initial strength. Oxidation, 1.0 mM H_2_O_2_, 60 min; Restore, 1.0 mM VC, 120 min. Reproduced with permission 124. Copyright 2019, American Chemical Society.

Sulfur, a typical non-metallic element, is prone to oxidation. This characteristic is also reflected in sulfur-containing polymers, which are prone to oxidation in ROS-rich environments. For example, sulfur atoms in thioethers are easily oxidized by ROS to form sulfoxide groups. This transformation often changes materials from hydrophobic to hydrophilic. The significantly enhanced water solubility enables DDS to achieve controllable drug release in ROS-enriched environments 120. Based on this principle, Liu *et al.* 121 developed the copolymer PEG-PPMT with pH and ROS response properties via amino groups and sulfide moieties. The copolymer self-assembled into nanocarriers in water and successfully encapsulated the chemotherapy drug, docetaxel (DTX). Experiments showed that the carrier structure disintegrated when sulfides were oxidized to sulfoxides in the TME, facilitating the specific release of DTX. This intelligent delivery system demonstrated excellent tumor-suppressing effects in *vivo* (Figure 8E-G), particularly showing precise and targeted therapeutic potential in tumors with elevated ROS levels. In a similar research direction, Wang's group 122 designed sulfide-based block copolymers based on polyethylene glycol (PEG) and poly (2-methyl-5-sulfostyrene). This amphiphilic polymer formed ROS-responsive nanoparticles via self-assembly (Figure 8A-E). Cell experiments have shown that nanocarriers can release loaded drugs in response to intracellular ROS.

Similarly, polymers with ROS-responsive groups have found wide application in tumor treatment. ROS at the tumor site can break thioketal bonds, thereby facilitating drug release. Ng *et al.* 123 first obtained a dimer by connecting 3,4-dihydroxy-L-phenylalanine to thiol groups (Figure 6 L-DOPA dimer). This dimer underwent self-polymerization in the presence of DOX to obtain DOX-containing polydopamine (PDA) nanoparticles, which were coupled with ZnPc photosensitizers and heptapeptides (QRH) capable of targeting the epidermal growth factor receptors to prepare the ROS-responsive polydopamine nanoparticles. After entering the cells through receptor-mediated endocytosis, these nanoparticles gradually degraded in the presence of ROS, releasing DOX and Pc molecules (Figure 8B-D). Under light exposure, they could generate more ROS, further promoting the degradation of nanoparticles and drug release, thereby forming a synergy between chemotherapy and photodynamic therapy. In nude mice with overexpression of EGFR-bearing tumors, PDA-Dox-Pc-QRH achieved tumor-targeted delivery, effectively inhibited tumor growth under light, and even resulted in complete tumor ablation.

**Figure 9:**
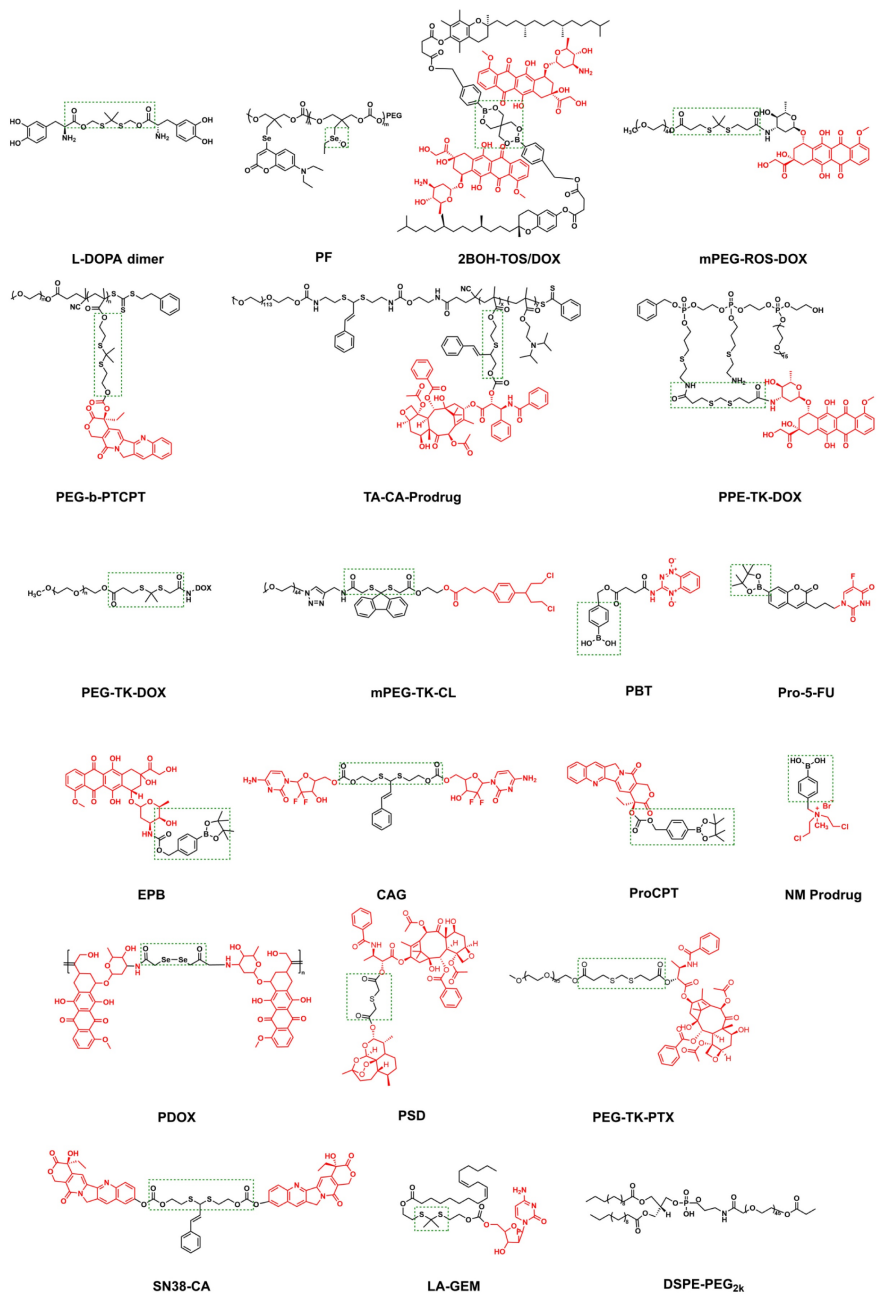
The structure of polymeric nanoprodrugs, the chemotherapy drug (highlighted in red) with a ROS-reactive group (in the green box).

As a sulfur homolog, selenium can also undergo specific oxidation reactions in the ROS microenvironment. The hydrophobic monoselenium group is oxidized to a hydrophilic selenosulfone group, which triggers drug release. Additionally, diselenide bonds can undergo oxidative breakage to form seleninic acid. This unique oxidative response mechanism offers a novel approach for tumor-targeted therapy. Li *et al.* 124 synthesized ROS-responsive nanoparticles PF using selenium-containing amphiphilic block copolymers and co-loaded cisplatin and paclitaxel (PTX) as chemotherapy drugs while introducing coumarin-based fluorescent groups (Figure 9 PF). The coordination effect of Se-Pt significantly improved the stability of the drug-loaded system while maintaining its high sensitivity to oxidative microenvironments. By leveraging the reversible transformation characteristics of selenide/selenosulfone in the selective redox cycle, simultaneous monitoring of the morphological changes of nanoparticles and the response of fluorescence signals was achieved (Figure 8F-M). The in *vitro* release behavior showed that the cumulative release amounts of PTX and cisplatin at 24 h without stimulation were less than 7% and 2.5%, respectively. After adding 1.0 mM H_2_O_2_, the cumulative release percentage of cisplatin within 12 h was 12%-17%, which was much lower than that of PTX (42%-51%). This might be due to the coordination of cisplatin with Se, which made it difficult to dissociate from the polymer chain. This integrated diagnostic and treatment design enables the precise release of drugs within tumor cells, which minimized harmful side effects in healthy tissues.

In addition to the above types, articles have reported the use of boronate crosslinking groups to prepare ROS-responsive nanomaterials. Wu *et al.* 125 prepared a novel α-TOS dimer through the crosslinking of phenylborate esters. The hybrid nanomedicine 2BOH-TOS/DOX was co-assembled from α-TOS dimers and DOX (Figure 9). Hydrophobic interactions and π-π stacking improve the efficiency of drug loading; additionally, controlled release of the drug can be achieved via the cleavage of boronate esters by H_2_O_2_. When H_2_O_2_ was not added, the release rate was significantly restricted, with less than 10% total release within 72 h, demonstrating that it could stably load the drug in a physiological environment and effectively prevent drug leakage or sudden release. The hydrolysis of boronate esters with H_2_O_2_ is typically a second-order reaction. The rate-determining step is the attack of the boron atom by H_2_O_2_, which acts as a nucleophile. The rate of this step is directly related to the concentration of both reactants. After H_2_O_2_ treatment, the release rate of DOX significantly increased. With an increase in H_2_O_2_ concentration (0.1mM-1.0 mM), the cumulative release amounts reached 52.37% and 69.23%, respectively. The α-TOS component could downregulate intracellular ATP levels, reduce drug efflux, and increase drug concentration, thereby reversing tumor drug resistance.

### 3.2. Linking drugs to polymers via ROS-responsive bonds to construct polymer nanoprodrugs

To effectively address drug leakage issues in traditional encapsulation systems, drugs can be coupled to the polymer skeleton through ROS-sensitive chemical bonds to construct nanoprodrugs. He *et al.* 126 developed a polyethylene glycol-doxorubicin (mPEG-ROS-DOX) prodrug based on thioketal linkers (Figure 9). This prodrug could be cleaved by ROS in the TME to release DOX. Experiments demonstrated that the prodrug significantly enhanced the drug's half-life and tumor accumulation efficiency in mice with HepG2 tumors by triggering apoptosis in tumor cells, suppressing proliferation, and facilitating necrosis of the tumor cells. Simultaneously, it significantly reduced systemic toxicity and achieved an efficient antitumor effect.

Researchers primarily adopt the strategy of constructing positive feedback ROS-responsive systems to achieve continuous prodrug release post-activation by ROS in cancer cells, thereby realizing the stepwise amplification effect by integrating redox-active molecules or introducing light-responsive dyes or photosensitizers 127. For example, β-Lapa, an NAD(P)H: NQO1-activating drug, exerts a dual effect by inducing tumor DNA damage and increasing ROS levels in tumor tissues 128. Ge *et al.* 129 created a novel delivery system utilizing this approach. Initially, they obtained an amphiphilic block copolymer prodrug consisting of PEG and polymethacrylate monomers; then, camptothecin (CPT) was coupled with a thioketal linker and self-assembled into core-shell micelles, PEG-b-PTCPT (Figure 9). Finally, Lapa was encapsulated to construct the composite nanoparticles (Lapa@NPs), which gathered at the tumor in *vivo* experiments. Simultaneously, Lapa selectively elevated the expression of NQO1 in the malignant cells, resulting in the generation of massive amounts of ROS. Elevated ROS levels triggered the rupture of the thioketal linker, thereby releasing CPT and forming a virtuous cycle of “ROS generation-drug release.” The released CPT acts in concert with the continuously elevated ROS, significantly inhibiting tumor growth through dual mechanisms: inhibition of topoisomerase I and induction of oxidative stress.

The TA-CA-Prodrug (Figure 9) developed by Luo *et al.* 130 used cinnamaldehyde (CA) as a ROS-responsive thioneone linker to achieve precise coupling of PTX to the copolymer's backbone. A pH-sensitive group, dipropylamine (DPA, pKa≤6.2), was introduced. This prodrug formed micelles through self-assembly, exhibiting a particle size of about 150 nm and a negative ζ-potential. In the acidic conditions prevalent in tumor cells, the protonation of DPA triggered charge inversion (from negative to positive), allowing it to target the negatively charged mitochondrial membrane through electrostatic interactions. The synergistic effect of CA-mediated ROS generation and mitochondrial localization significantly enhanced the specificity and efficiency of drug release. Based on this, Yuan *et al*. 131 performed functional expansion. They coupled the CA linker with DOX and the polymer skeleton, co-loaded the fluorescent prodrug BCyNH_2_, and self-assembled to form the polyprodrug PEG-TA-CA-DOX, which could be degraded by ROS. Upon entry into the cancer cells, the prodrug was activated by endogenous ROS, resulting in the liberation of a small quantity of bioactive drug. The released CA and CyNH_2_ induced ROS production through mitochondrial dysfunction, consequently hastening the collapse of PTCD@B and triggering prodrug release.

**Figure 10:**
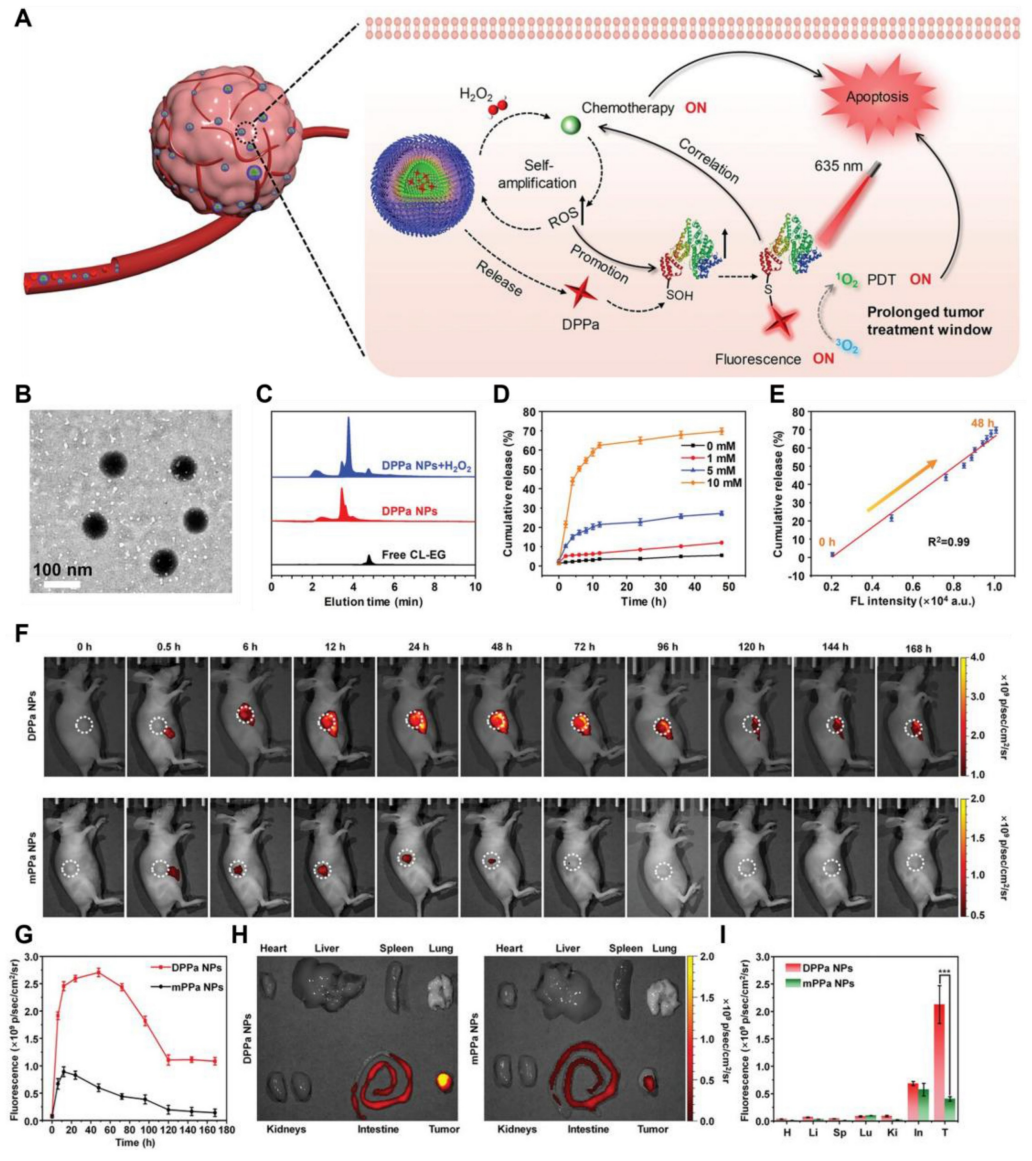
(A) Schematic illustration of fluorescence imaging-guided combined chemo/photodynamic therapy using DPPa NPs to enhance the therapeutic window. (B) TEM image depicting DPPa NPs. (C) HPLC analysis of DPPa NPs (10 μm) following a 1-hour incubation with 1 mM H_2_O_2_ at 37°C for 1 hour. (D) In *vitro* release characteristics of CL-EG of 1 mM DPPa NPs in PBS at 37°C (pH = 7.4) under different concentrations of H_2_O_2_. (E) A linear relationship between the fluorescence intensity of DPPa NPs and the release rate of CL-EG. Error bars indicate the standard deviations from three separate measurements. (F) Temporal fluorescence imagery of tumor-bearing mice post-intravenous administration of either DPPa NPs or mPPa NPs (100 μL, 500 μg/mL) (λex = 640 nm, λem =710 nm), with the tumor location indicated by a white circle. (G) Changes in fluorescence intensity within the tumor over time following the injection. (H) Representative images of the major organs and tumors (T = tumor, H = heart, Li = liver, Sp = spleen, Lu = lung, Ki = kidney, In = intestine) of mice 7 days after intravenous injection of DPPa NPs or mPPa NPs. (I) Near-infrared fluorescence intensity. The error bar represents the standard deviations of three different measurements (n = 3), with ***p < 0.001. Reproduced with permission 135. Copyright 2023, Wiley-VCH GmbH.

Spatiotemporally controllable release systems mediated by photosensitizers have become a research hotspot in recent years. Park *et al.* 132 constructed a hydrophilic PEG-DOX conjugate (PEG-TK-DOX) by linking DOX and PEG through thioketal bonds (Figure 9). PEG-DOX could self-assemble into nanoparticle systems with biological activity and ROS responsiveness. DOX release was triggered by ROS generated by the photosensitizer PhA, achieving chemotherapy-photodynamic synergistic therapy. Wang *et al.* 133 co-assembled PPE-TK-DOX (Figure 9) with Chlorin e6 (Ce6) to form nanoparticles (Ce6@PPE-TK-DOX), and utilized the photosensitivity of Ce6 to enhance ROS generation efficiency. Fluorescence imaging conducted in *vivo* revealed that the fluorescence signal was widely distributed throughout the mouse body within 1 h post-injection. Thereafter, the strength of the fluorescence signal from Ce6 at the tumor location gradually rose and peaked at 4 h post-injection. Wang *et al.* 134 also fabricated nanomaterial PSPC NAs using this design concept. Under near-infrared (NIR) light irradiation, photosensitizers generated ROS that spontaneously degraded thioketal bonds and released the drug. Fan *et al.* 135 developed the H_2_O_2_-activated self-expanding photodynamic/chemotherapy combination therapy drug DPPa NPs. Hydrophobic oxidized bovine serum albumin (BSA-SOH) conjugated with DPPa NPs encapsulated mPEG-TK-CL, an amphiphilic prodrug activated by H_2_O_2_, along with chlorambucil (CL). (Figure 9). DPPa NPs achieved fluorescence signal recovery and photodynamic effect amplification through BSA-SO_3_H coupling, and their macromolecular structure significantly prolonged the tumor residence time (Figure 10). This study provided a reference strategy for prolonging the PDT window period. To address the key challenge of poor permeability of tumor nanomedicines, Bai *et al.* 136 developed a nanomedicine by natural mussel adhesion protein (NMPs), which was conjugated with tilapazine (TPZ) and 4-(hydroxymethyl) phenylboric acid succinic anhydride to form a phenylboric acid prodrug (Figure 9 PBT). After mixing PBT with NMP and doping it with ICG, they obtained ICG-PBT@NMPs nanomedicine. In the TME, the positive charge characteristics were reversed, promoting tumor penetration. Subsequently, tumor cells internalized ICG-PBT@NMPs via endocytosis mediated by arginine transporters. Under near-infrared irradiation, the ICG-PBT@NMPs generated ROS, exacerbating tumor hypoxia and enhancing PBT activation. The design concept based on NMP in this study could be extended to the design of other drugs.

**Figure 11:**
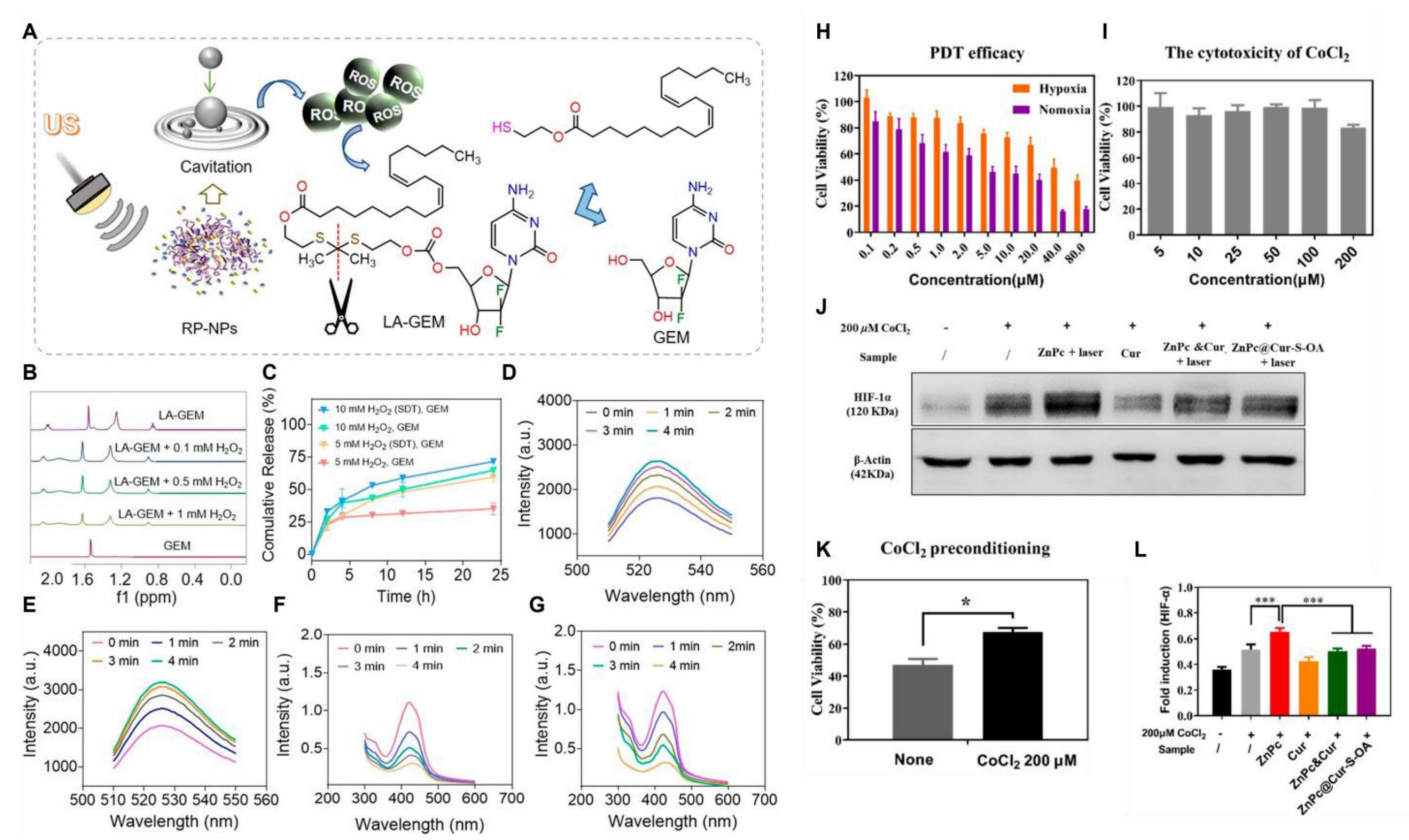
(A) Schematic diagram of GEM release after thione bond cleavage induced by US irradiation. (B) ^1^H NMR spectra of LA-GEM solution treated with different concentrations of H_2_O_2_. (C) Release curves of GEM under irradiation with different concentrations of H_2_O_2_ or US (n = 3). (D, E) Fluorescence intensities of SOSG solution (5 μM) after incubation without (D) or with (E) RP-NPs and then prolonged US irradiation. (F, G) Ultraviolet-visible spectra of DPBF solution (0.1 mg/mL) after incubation without (F) or with (G) RP-NPs followed by prolonged US irradiation. Reproduced with permission 137. Copyright 2023, American Chemical Society. (H) Cell viability of B16F10 cells after PDT treatment with different concentrations of ZnPc under normoxic or hypoxic conditions. (I) Cytotoxicity of different concentrations of CoCl_2_ against B16F10. (K) After incubating B16F10 cells with 200 μM CoCl_2_ for 24 hours, PDT treatment with 10 μM ZnPc was performed to determine the cell viability (N = 12). (J, L) HIF-1α protein levels were determined by Western blot. (N = 3). *, P < 0.05; ***, P < 0.001. Reproduced with permission 138. Copyright 2020, American Chemical Society.

Noninvasive sonodynamic therapy (SDT) activates sonosensitizers accumulated at tumor sites using ultrasound and stimulates the production of large amounts of ROS. Its tissue penetration ability is stronger than that of PDT. Wan *et al.* 137 constructed a ROS-triggered self-assembled nanoparticle (RP-NPs). RP-NPs were composed of the prodrug LA-GEM (Figure 9) (gemcitabine prodrug with thioketal linker), the natural sonosensitization agent rhein (Rh) (Figure 6), and DSPE-PEG_2k_ (Figure 9). During ultrasound (US) irradiation, Rh was activated and generated a large amount of ROS via acoustic cavitation (Figure 11A). On the one hand, it directly induced apoptosis in the tumor cells. Conversely, it triggered the cleavage of the thioketone bond of LA-GEM, resulting in the targeted and accelerated release of GEM within the tumor (Figure 11B-G). The US could increase the permeability of tumor tissues, improve the hypoxic microenvironment, and synergistically enhance the effect of chemotherapy. SDT could control the treatment range by adjusting ultrasound parameters (e.g., frequency and intensity) and has fewer local side effects (e.g., skin damage). Its biocompatibility is superior to that of PDT and radiotherapy.

All the aforementioned self-amplifying nanomedicine systems initiate a cascade reaction by generating ROS via photosensitizers or drugs. The downregulation of GSH disrupts the antioxidant defense system of tumor cells. When ROS activates the prodrug, tumor cells become more vulnerable to drug-induced attacks, thereby overcoming drug resistance 139. Curcumin (Cur), a natural medicinal component isolated from turmeric, can significantly reduce Hypoxia-Inducible Factor 1α (HIF-1α) levels and consume GSH in various tumor cells 140. Qu *et al.*^138^ designed and fabricated multifunctional combination therapy nanoparticles ZnPc@Cur-S-OA with both self-delivery and self-monitoring capabilities, using Cur as a chemotherapy drug and ZnPc as a photosensitizer. The ROS-activated Cur prodrug (Cur-S-OA) achieved PDT-enhanced cancer treatment by decreasing HIF-1α (Figure 11H-L). The “OFF-ON” activation presented by the green fluorescence of Cur during this process was utilized to monitor drug release.

### 3.3. Stimuli-responsive materials encapsulate ROS-responsive small molecule prodrugs to construct polymer nanoprodrugs

The polymer nanoprodrugs mentioned above can only respond to a single stimulus, ROS. However, adapting a single response mechanism to the diverse and dynamic changes in the TME is challenging 141. The pH values, enzyme activities, redox states, and other aspects of the TME between different genres of tumors and the same tumor in different stages of development may be discrepant. It is difficult for drugs with a single response mechanism to cope with these changes, leading to suboptimal specific enrichment and release effects of these prodrugs in tumor tissues, ultimately affecting their therapeutic efficacy 142. Many recent studies have utilized polymer nanomaterials that can be decomposed under tumor-specific triggering factors (e.g., GSH, pH, and enzymes) as carriers to encapsulate small-molecule prodrugs that can be activated by ROS, thereby constructing dual-responsive DDS 143. After the outer layer of nanomaterials is disrupted in the tumor tissue, the encapsulated small-molecule prodrugs are released. These small-molecule prodrugs are further activated by ROS, releasing the chemotherapeutic drugs. The release time and rate of drugs can be more accurately controlled by the dual-response mechanism, thereby better meeting the precise requirements for drug release in tumor therapy.

The high GSH levels in tumor cells can serve as a specific trigger signal in conjunction with ROS. GSH consumption weakens the antioxidant defense of tumor cells, rendering them more susceptible to ROS. In contrast, ROS generation further consumes GSH. These two processes promoted each other, synergistically inducing tumor cell death more effectively and enhancing the antitumor effect of the prodrugs. Zhang *et al.* 144 developed novel cross-linked lipoic acid nanocapsules (cLANCs) connected by disulfide bonds using (R)-(+)-lipoic acid (LA) as the raw material. Both LA and its reduced state (lipoic acid hydride, DHLA) could act as pro-oxidants to increase the generation of ROS within cells. Due to their structural autoploidy with LA, the cross-linked lipoic acid nanoparticles exhibited good biocompatibility and could serve not only as drug carriers but also as pro-oxidants to elevate ROS levels. The ROS-sensitive prodrug, Pro-5-FU (Figure 9), was loaded into H_2_O_2_-amplifying cLANC to develop the nanoprodrug Pro-5-FU@cLANCs. After entering the cancer cells, they were destroyed by GSH, and LA was released. This process increased the H_2_O_2_ levels in nanomedicine-treated tumor cells to 3.4 times higher than those in untreated tumor cells, thereby accelerating the release and activation of the prodrug, Pro-5-FU. Gan *et al.* 145 co-delivered the ROS-activated prodrug EPB (Figure 9) and the highly efficient NQO1 substrate KP372-1 by adhesive nanocarriers responsive to GSH. KP372-1 was more effective than existing NQO1 substrates, and a little KP372-1 in NPs exerted a powerful effect on ROS generation but did not exhibit cytotoxic effects. The dual activation of these nanoparticles significantly broadened the selection window between normal and tumor cells.

The low pH of the TME is also widely used as a trigger for the development of nanoparticles. Yang *et al*. 146 constructed pH-responsive micelles through self-assembly using pH-responsive block polymers PEG-Hyd-PCL and cationic block polymers PEI-PCL. Subsequently, these micelles were used to coat the ROS generator β-Lap and the DOX prodrug, BDOX, to form PHI@B/L. The acidic environment in the tumor triggered the cleavage of hydrazone bonds, resulting in the exposure of the positively charged layer and increased uptake of the drug by tumor cells. After the nanosystem was internalized, Lap and BDOX quickly escaped from lysosomes. β-Lap could mediate the generation of ROS, thereby activating the prodrug BDOX and promoting its activation. Furthermore, β-Lap simultaneously consumed ATP, thereby reducing the energy supply of the drug efflux pump and promoting the reversal of multidrug resistance (MDR). This study provided a new perspective for studying the molecular mechanisms in cancer therapy.

Zhao *et al.* 147 synthesized a ROS-activated self-destructing prodrug CAG (Figure 9) using CA and the chemotherapy drug GEM as raw materials. With the help of G≡C-type hydrogen bonding interactions, CAG could efficiently bind to the guanine-rich acyclovir-modified hyaluronic acid conjugate HA-ACV and form the supramolecular nanoprodrug HCAG through self-assembly. After injection, the HCAG accumulated at the target tumor site. Subsequently, the acidic environment in the lysosome disrupted the hydrogen bonds, similar to base pairing (G≡C) inside the HCAG, causing the structure to disintegrate and the rapid release of free CAG. ROS in tumor cells first activated a small amount of CAG, releasing CA and GEM. CA promoted the production of ROS, which in turn activated the remaining CAG, thereby establishing a self-reinforcing positive feedback loop. Therefore, the HCAG nanoformulation effectively targeted tumors and improved the biodistribution and accumulation of CAG in tumors. Ge *et al.* 148 synthesized the copolymer CAMA-co-ImOAMA using the unstable acetal bond and CA, which was then self-assembled into pH-responsive polymer micelles, and the ROS-responsive prodrug pinacol phenylboronate caged CPT was loaded (Figure 9 ProCPT). The PIMOAMA fragment within the micelles provided nanoparticles with enhanced endosomal escape ability. When micelles ruptured in the tumor cell endosome owing to the acidic environment, the PIMOAMA fragment could breach the endosomal membrane, allowing the released free CA and ProCPT to enter the cytoplasm. CA might increase intracellular ROS levels, thereby enhancing ProCPT activation efficiency. The methylquinone produced during prodrug activation reduced intracellular GSH levels and exerted a synergistic antitumor effect. Zhao *et al.* 149 acetalized maltose (MH) to obtain the pH-sensitized hydrophobic fragment AcMH. Subsequently, a click reaction linked the hydrophilic segments PAsp and mPEG to form amphiphilic block polymers PAsp-AcMH and mPEG-AcMH. mPEG-AcMH and PAsp-AcMH self-aggregated with the nitrogen mustard (NM) prodrug (Figure 9) and Lapa to form nanoparticles. Following intravenous injection, these nanoparticles could localize at the tumor site. The weakly acidic environment caused the acetal bond to break and released the NM prodrug and Lapa. Lapa induced the production of a large amount of H_2_O_2_, which further activated NM prodrug. The control of precise drug release by dual-responsive nanoprodrugs was primarily reflected in the “silencing” of non-target environments, the “specific activation” of target environments, and the “synergistic filtering” effect of dual signals. Liu *et al.* 150 linked DOX to the polymer main chain *via* an acid-labile hydrazone bond and a diselenide (Figure 9 PDOX). The release of DOX required simultaneous satisfaction of two conditions: low pH and a high level of ROS. In a medium with a pH of 7.4, even at a GSH concentration of 10 mM (far exceeding the level in normal tissues), drug release was negligible. Meanwhile, at pH 5.0 of tumor areas, but with a GSH concentration of zero or low level (0.1 mM, non-tumor feature), the drug is also hardly released. The drug showed detectable cumulative release (10.3% and 7.4% within 96 h) only at pH 5.0 (acidic) and in the presence of high concentrations of GSH (10 mM) or H_2_O_2_ (0.5 mM). The “dual filtering barrier” not only avoided the accidental leakage of drug molecules in nanomedicines designed through only one controllable condition but also significantly improved the drug-loading capacity of nanocarriers.

### 3.4. Iron-coordinated nanocarriers encapsulating small molecule prodrugs to construct polymer nanoprodrugs

**Figure 12:**
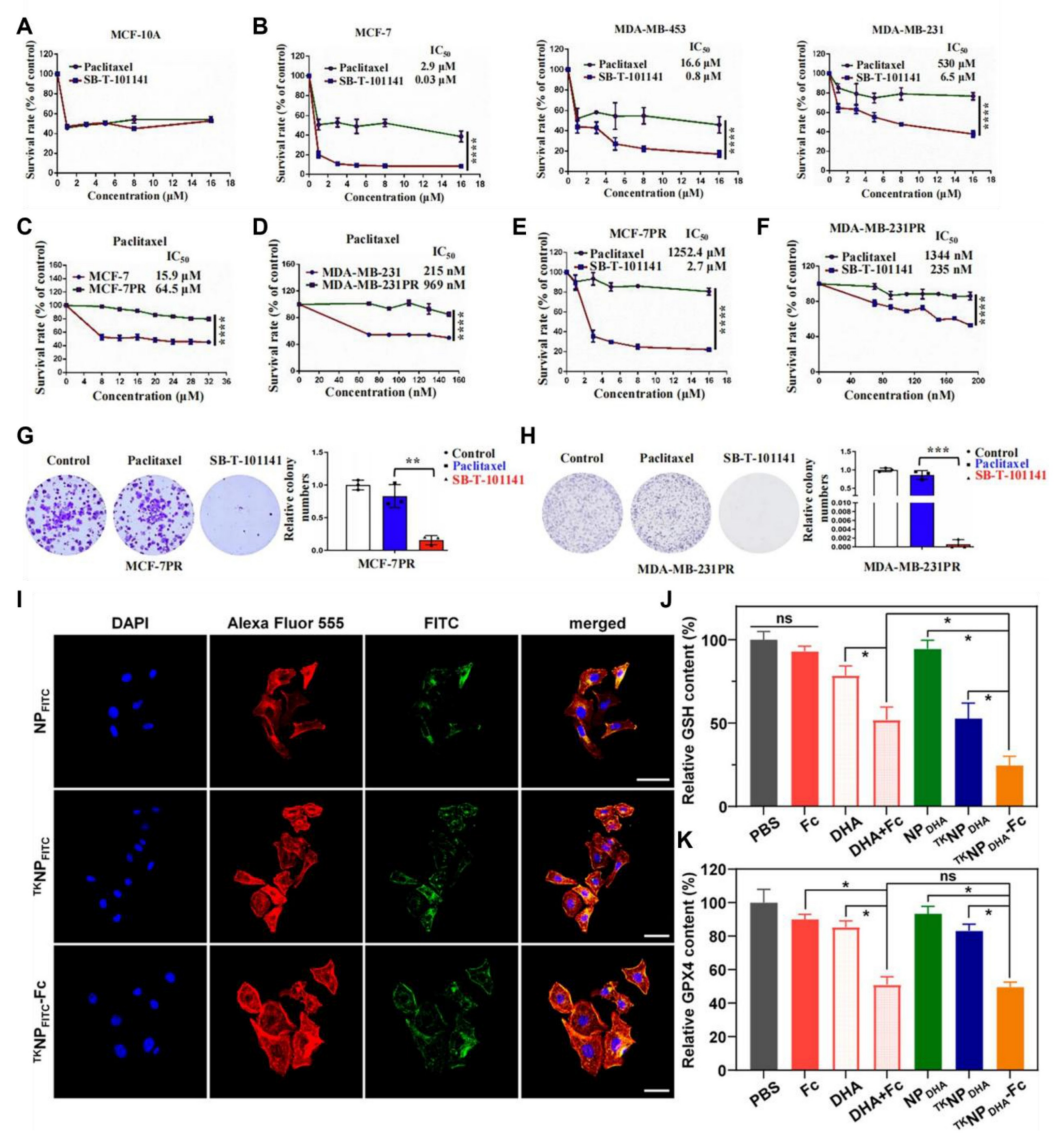
(A, B) Cell viability analysis of different cell lines after 72 hours of treatment with paclitaxel and SB-T-101141 at specified concentrations. (C-F) Viability analysis of drug-resistant cells (MCF-7PR, MDA-MB-231PR) and non-drug-resistant cells (MCF-7, MDA-MB-231) 72 hours after administration. Results were analyzed using a Two-way *ANOVA* test (mean ± SD, n = 3). *P < 0.05, **P < 0.01, ***P < 0.001, and ****P < 0.0001. (G, H) Crystal violet staining after MCF-7PR and MDA-MB-231PR cells were treated with paclitaxel or SB-T-101141. Results were analyzed using a Student's t-test (mean ± SD, n = 3) (right panel). *P < 0.05, **P < 0.01, ***P < 0.001, ****P < 0.0001. Reproduced with permission 154. Copyright 2025, Springer Nature. (I) CLSM observation of 4T1 cells treated with FITC-loaded NP, ^TK^NP, or ^TK^NP-Fc. The scale bar is 50 μm. (J, K) Relative glutathione level and content of GPX4 in 4T1 cells upon incubation with different formulations (mean ± SD). Fc equivalent concentration was 10 μΜ. ns, no significant differences. *p < 0.05. Reproduced with permission 155. Copyright 2024, American Chemical Society.

Fe²⁺ and Fe³⁺ can generate ROS by Fenton or Fenton-like reactions 151. Fe²⁺ can also reduce H₂O₂ to OH•, thereby causing cell damage 152. Tumor cells highly express the transferrin receptor (TfR1), which absorbs more iron to meet their rapid proliferation requirements. Introducing iron into prodrugs exploits the high iron uptake characteristics of tumor cells, enabling prodrugs to enter tumor cells more effectively through TfR1-mediated endocytosis 153. Therefore, previous studies have utilized iron-coordinated nanocarriers to deliver small-molecule prodrugs activated by ROS. Chemotherapeutic drugs often used in combination with iron-coordinated nanocarriers include PTX and dihydroartemisinin (DHA). Research indicates that inducing ferroptosis in cancer cells can overcome their resistance to paclitaxel therapy. For instance, innovative taxane SB-T-101141 initiated ferroptosis through an iron-stable-related mechanism involving KHSRP, thereby overcoming paclitaxel resistance in breast cancer (Figure 12A-H) 154.

Yin *et al.* 156 linked PTX and DHA through a thioether bond to synthesize a prodrug that could be activated by ROS (Figure 9). This prodrug self-assembled to form PTX-S-DHA nanoparticles, which exhibited a significant capacity for drug loading. While tumor cells exhibited a greater concentration of H_2_O_2_ compared to normal cells, it was easily consumed, thereby limiting the number of OH• produced by the Fenton reaction. Therefore, the self-assembly prodrug PSD was co-precipitated with PEG2000-ferrous iron (Fc) to create nanoparticles known as PSD-Fc nanoparticles. In the presence of Fe^2+^, DHA exhibited catalytic activity resembling that of a peroxidase, which promoted the Fenton reaction within cells, resulting in the production of a significant quantity of ROS. The synergistic effect of PTX led to the death of tumor cells. To achieve effective co-transport of iron toxicity inducers, exogenous ferrous ions, and chemotherapeutic drugs. Similarly, Zhang *et al.* 155 coupled PTX with ferrocene (Fc) using thiophenone to prepare the DHA-loaded nanocells (^TK^NP_DHA_-Fc) (Figure 9 PEG-TK-PTX). Through the synergistic effects of chemotherapy and ferroptosis, significant antitumor effects were demonstrated both in *vivo* and in *vitro* (Figure 12I-J). Kamei *et al*. 157 prepared nanoparticles SN38-CA@FC NPs (Figure 9 SN38-CA) using a one-step nano-precipitation method. They linked the CPT derivative SN38 to the ROS generator CA via thioacetone and co-assembled it with Fc. SN38-CA@FC nanoparticles induced the accumulation of lipid peroxides (LPOs) by consuming GSH and GPX4, ultimately triggering severe oxidative damage and cell death. This intelligent precursor nanosystem, composed of ferroptosis inducers and chemotherapy drugs, maximized the effects of ferroptosis and chemotherapy by leveraging the Fenton reaction.

## 4. ROS-Triggered Hydrogel Prodrugs

**Figure 13:**
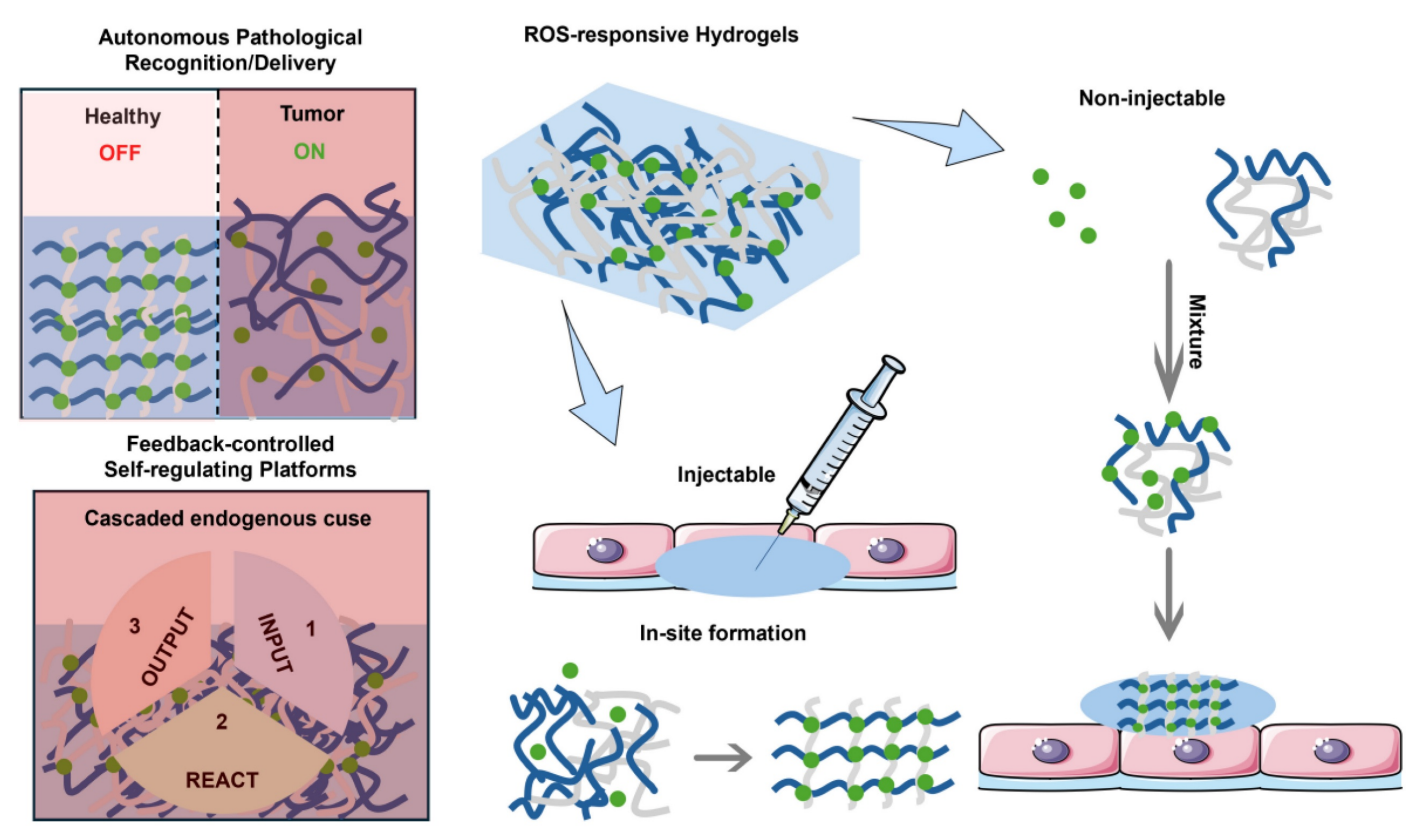
The drug release mechanism of hydrogels triggered by ROS: Two types of ROS-responsive hydrogels (injectable and non-injectable) have achieved site-specific drug release by taking advantage of the ROS in the TME, and have the benefits of local retention, controllable release, and multimodal treatment.

As a hydrophilic substance, hydrogels have become an essential supplement to nanoparticle DDS because of their safety to normal tissues 158. Compared to nanoparticle carriers, hydrogels offer unique advantages in drug delivery, particularly with broad application prospects in cancer treatment. Over the past few years, hydrogel DDS have evolved from passive release to intelligent controlled-release systems that can react to diverse external stimuli, including pH, ROS, heat, light, and ultrasound, and have further developed in the direction of multi-responsive collaborative regulation 159. Among them, ROS-responsive hydrogels can achieve targeted drug release by introducing specific reaction units that cause hydrophilic and hydrophobic transitions of polymer chains or break chemical bonds in a high ROS environment 12,160. This section focuses on discussing this type of drug delivery hydrogel systems responsive to ROS through solubility conversion or bond cleavage mechanisms (Figure 13).

### 4.1. Hydrogels with solubility switch units

**Figure 14:**
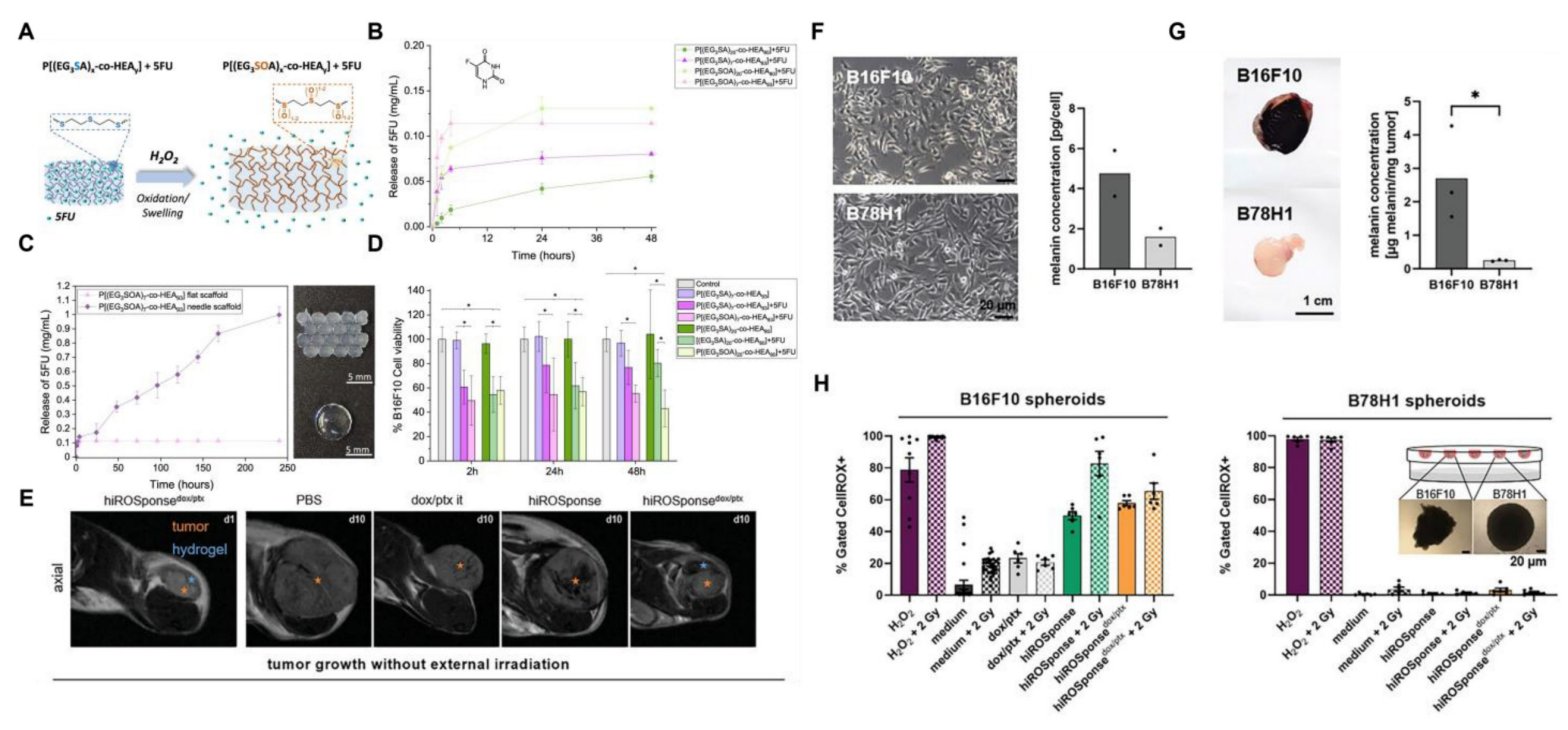
(A) Schematic depiction of the release of 5FU from P[(EG_n_SA)_x_-co-HEA_y_] hydrogels, which is facilitated by oxidation and swelling in a system that contains H_2_O_2_. (B) Release of 5FU from hydrogels (P[(EG_3_SA)_20_-co-HEA_80_] and P[(EG_3_SA)_7_-co-HEA_93_]) was examined under non-oxidative conditions in PBS as well as under oxidative conditions with the inclusion of 9 mM H_2_O_2._ (C) The two types of P[(EG_3_SOA)_7_-co-HEA_93_] hydrogels (needle scaffold and flat surface scaffold) release 5FU when subjected to oxidizing conditions (1 mM H_2_O_2_). (D) Evaluation of P[(EG_3_SA)_20_-co-HEA_80_] and P [(EG_3_SA)_7_-co-HEA_93_] release of 5FU of B16F10 in vitro cytotoxicity. One group was under non-oxidizing conditions, where cells were co-cultured with hydrogels in PBS. Another group was under oxidation conditions, with an additional 1 mM H₂O₂ added to the co-culture system. All experimental data were expressed as “mean ± standard deviation” (sample size n=3), and the Tukey test was used for analysis of variance, with the significance level set at *p < 0.5. Reproduced under terms of the CC BY 4.0 license 161. Copyright 2023, by Miryam *et al*. (E) Representative magnetic resonance imaging (MRI) of subcutaneous solid B16F10 tumor (orange mark) and the injected hydrogel (highlighted in blue) are shown at day 1 and day 10 post hiROSponse injection. (F) The structure of cells and the amount of melanin in melanotic B16F10 and B78H1 cells when grown in a monolayer culture. (G) Samples of B16F10 and B78H1 tumors were representative, and melanin levels were quantified in the excised tumor specimens. A two-tailed unpaired t-test was utilized for statistical evaluation, with a significance threshold set at *p < 0.05 and a sample size of n=3. (H) Flow cytometry was employed to measure the levels of ROS in spheroids of B16F10 and B78H1. Cells were selected based on the fluorescence signals that were positively emitted by the CellROX probe. The range of sample sizes varied from n=5 to n=29, with all results presented as mean ± standard error. An image is provided depicting the microscopic observation of B16F10 and B78H1 spheroids after a culture period of four days utilizing the hanging drop technique. Reproduced under terms of the CC BY 4.0 license 162. Copyright 2024, by Hauser *et al*.

Miryam *et al.* 161 have developed a novel water-soluble redox-responsive monomer based on ethylene glycol thioacrylate (EG_n_SA), which could be polymerized by ultraviolet light (UV) and form a 4D printable hydrogel exhibiting high-resolution properties. The antitumor drug 5-fluorouracil (5FU) was encapsulated in the hydrogel system. Notably, the sulfide groups in the EG_n_SA molecule changed to hydrophilic sulfoxide or sulfone groups under the action of H_2_O_2_ or hypochlorite, thereby triggering the expansion of the hydrogel network and facilitating controllable drug release (Figure 14A-D).

Compared with traditional preformed hydrogels, injectable hydrogels can be formed in situ using minimally invasive surgery, effectively covering areas that are hard to reach with traditional surgical approaches. Free chemotherapy drugs that are directly injected into the tumor site can be quickly cleared by the circulatory system. In contrast, hydrogel DDS can maintain a long-lasting local drug concentration in tumors. Based on this, Hauser *et al.* 162 constructed an injectable ROS-responsive hydrogel (hiROSponse) using Fc to co-load the melanoma treatment drugs DOX and PTX. Hydrophilic DOX was physically embedded in the hydrogel network, and its release followed the Fick diffusion mechanism. The hydrophobic drug PTX was loaded via hydrophobic interactions with Fc. In the microenvironment with a high redox potential specific to melanoma, Fe^2+^ was oxidized to Fe^3+^, resulting in the disruption of hydrophobic interactions, thereby achieving the specific release of PTX. In tumor studies, injection of PBS, dox/ptx, hiROSponse, hiROSponse^dox^, or hiROSponse^ptx^, hiROSponse^dox/ptx^ significantly slowed the tumor growth (Figure 14E-H).

Immunotherapy activates the immune system through the use of immunomodulatory drugs, antibodies, or adjuvants. However, the nonspecific biological distribution of immunotherapeutic drugs often leads to insufficient response rates and adverse immune reactions. It is essential to create nano-delivery systems capable of continuously releasing drugs in tumors. Chen *et al*. 163 have developed an innovative ROS-responsive injectable thermogel system. This system was based on a methoxy PEG block polysulfide copolymer (mPEG-b-PMet) co-loaded with the chemotherapy drug DOX, antiviral drug imidazoquinoline, immunomodulator R848, and immune checkpoint inhibitor αPD-1. The gel solution underwent a temperature-responsive sol-gel transformation after being injected into the tumor site. Its methionine methyl sulfide group was oxidized to sulfoxide units by ROS, triggering the regulated release of the drug. In the B16F10 melanoma mouse model, the hydrogel co-loaded with DOX/R848/αPD-1, when locally injected, not only suppressed tumor growth and extended survival but also elicited a robust anti-tumor immune response while maintaining low systemic toxicity. Notably, this system also demonstrated an outstanding ability to prevent recurrence and foster long-term immune memory effects, confirming the clinical translational potential of the hydrogel-based local chemoimmunotherapy strategy.

### 4.2. Hydrogels with cleavable units

In addition to the solubility transformation mechanism triggered by ROS, studies have reported the integration of fracture units that react with ROS into the hydrogel. This ROS-responsive hydrogel can control drug release by breaking the polymer chain under oxidizing conditions, ensuring that the hydrogel is completely degraded into nontoxic molecules and avoiding long-term retention caused by immune responses.

Hydrogels prepared from natural polymers exhibit remarkable biocompatibility, degradability, and flexibility, making them extensively employed in biomedical applications. Carboxymethyl cellulose (CMC), sourced from natural cellulose, presents outstanding development opportunities. CMC exhibits significant physical and chemical characteristics, including water absorption, film-forming ability, and low immunogenicity, in biological fluids. However, the low mechanical strength and rapid degradation limit their application in biomedicine. Therefore, they must be modified to obtain hydrogels with better mechanical properties and higher drug-loading efficiency. Lim's team 164 constructed a mechanically stable biomedical hydrogel through an inverse electron-demand bond reaction involving Tz and Nb. The nitrogen byproduct promoted the development of a porous structure within the hydrogel. The photosensitizer ICG and chemotherapy drug DOX were coated onto a porous network. Under near-infrared light irradiation, ICG generated ROS, broke the thioketal bond, disrupted the hydrogel structure, and released drugs. Some studies have also utilized ROS to induce the in-situ formation of hydrogels and bioorthogonal reaction-mediated prodrug activation. Gao *et al.* 165 developed a self-reporting system for the activation of bioorthogonal prodrugs. The Tz group serves as both a fluorescence quencher and a prodrug activator. When it underwent Diels-Alder (IEDDA) reaction with the prodrug TCO-PTX, which was modified with trans-cyclooctene (TCO), it relieved the fluorescence quenching. It released the active drug, and the fluorescence intensity is linearly related to the amount of drug released.

Li *et al.* 60 utilized the calcium-induced gel properties of alginate to construct an injectable in-situ formed hydrogel. The alginate solution contained PpIX-modified Fe_3_O_4_ nanoparticles (PF) and programmed death ligand 1 antibody (αPD-L1), which were linked to BSA using ROS-responsive linkers. The photosensitizer PpIX produced ^1^O_2_ under near-infrared light irradiation for photodynamic therapy. Fe_3_O_4_ could undergo a Fenton reaction to produce OH• and exert chemotherapeutic effects.

The production of large amounts of ROS could lead to immunogenic cell death (ICD) and disrupt thiol bonds, releasing αPD-L1. αPD-L1 bound to PD-L1, blocking tumor immune escape mediated by the PD-1/PD-L1 pathway and enhancing the killing activity of T cells against tumor cells. The hydrogel platform of this prodrug exhibited dual responses to the TME and near-infrared light, which prominently restrained the development of both primary breast cancer tumors and metastatic lesions and effectively prevented metastasis in the lungs and liver. Immunofluorescence staining indicated an elevation in the expression levels of calreticulin (CRT) and high-mobility group protein B1 (HMGB1) within tumor tissues following APPF+laser treatment, thereby promoting dendritic cells (DCs) maturation.

## 5. ROS-Triggered Inorganic Nanoprodrugs

**Figure 15:**
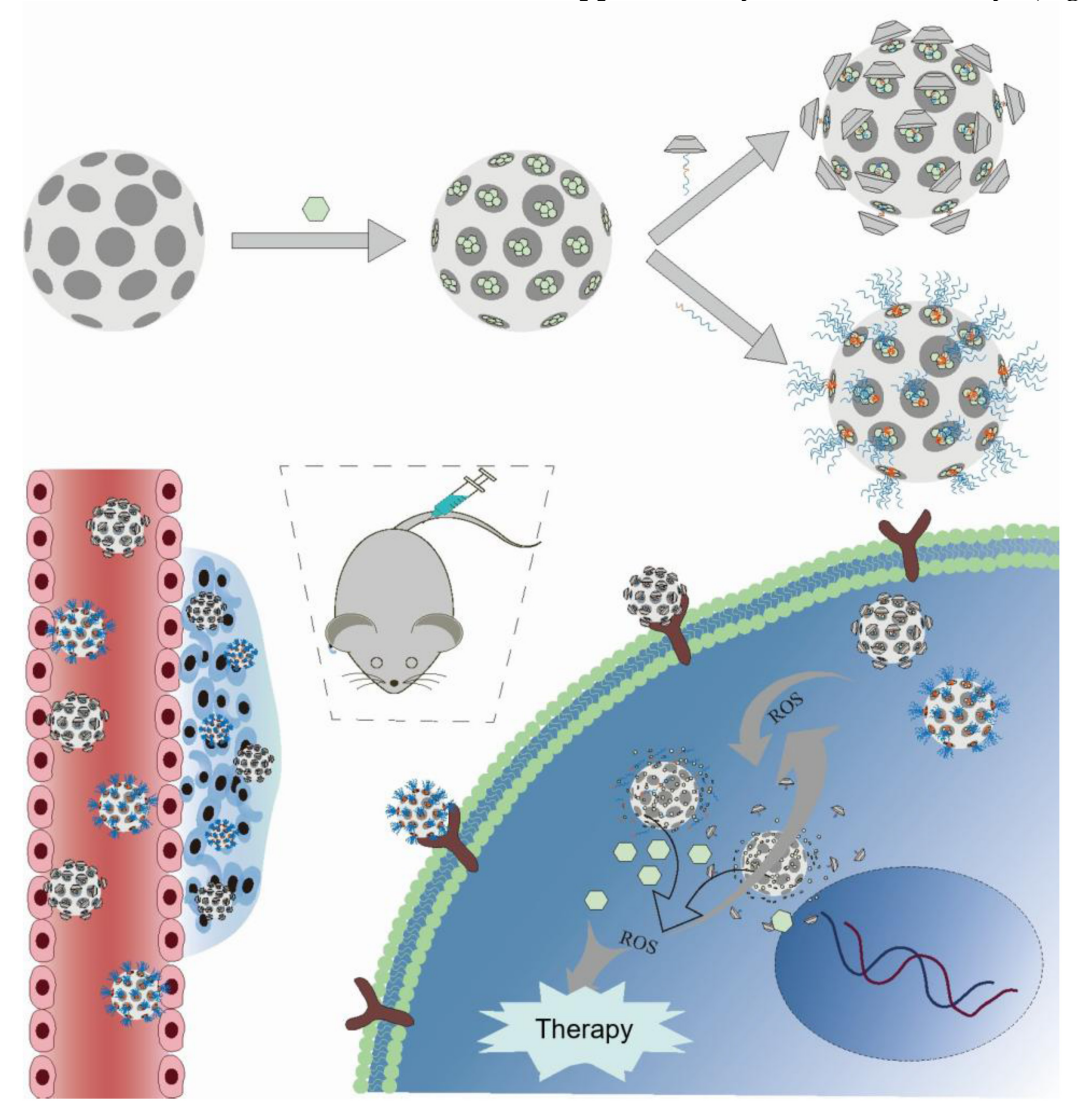
The ROS-triggered drug release mechanism of inorganic nanoparticles in tumor treatment. MSNs and MTNs are key inorganic carriers for ROS-responsive drug delivery. Their surfaces are linked to “gated molecules” through ROS-responsive linkers, preventing premature drug leakage. After functional modification, targeted drug release and imaging localization can be achieved. Meanwhile, tumor-targeting ligands are transplanted onto the surface of nanoparticles to enhance receptor-mediated endocytosis.

Inorganic nanoparticles include inorganic particles and biodegradable polycationic synthesis 166. Materials with mesoporosity exhibit elevated specific surface areas, significant pore volumes, and tunable mesoporous architectures and compositions. They have great potential in environmental protection, energy storage and conversion, biomedicine, and other fields 167. Among them, mesoporous titanium dioxide nanoparticles (MTNs) and mesoporous silica nanoparticles (MSNs) possess characteristics such as uniform pores, easy functionalization, elevated specific surface area, substantial pore volume, favorable biocompatibility, and biodegradability 168,169. In particular, MTNs and MSNs induce the generation of ROS in several cell lines, indicating their potential in creating DDS that respond to ROS. Thus, this section discusses such systems based on MTNs and MSNs (Figure 15).

**Figure 16:**
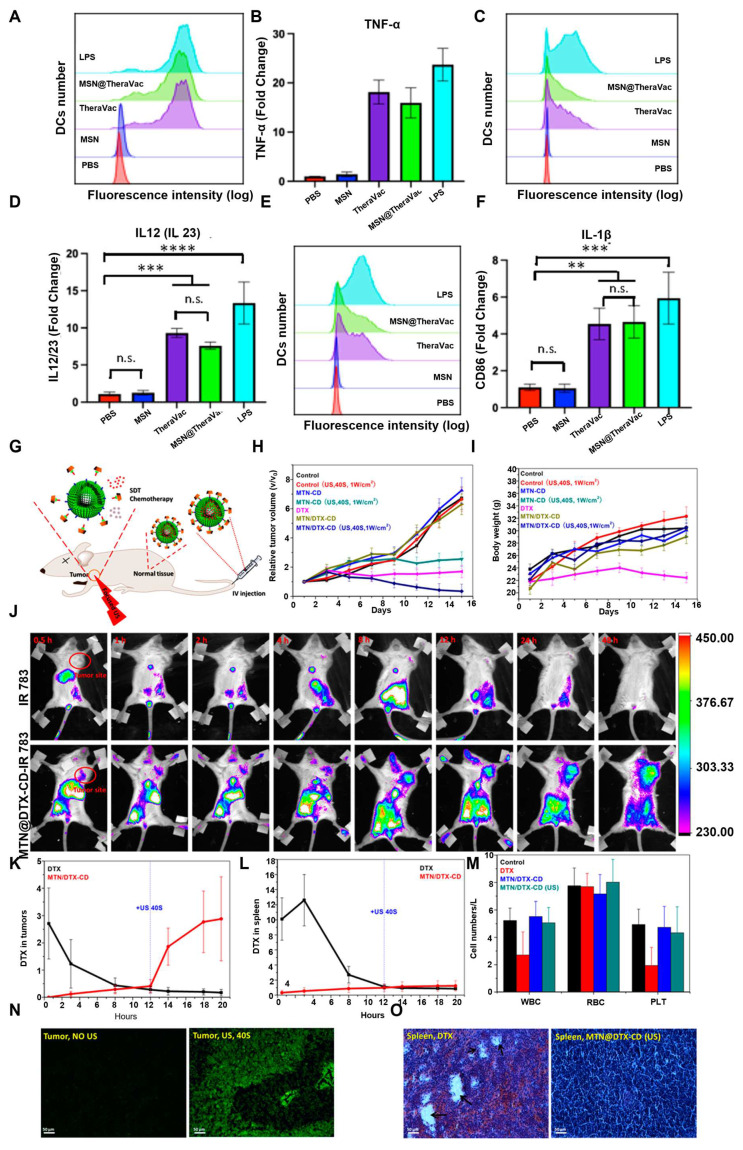
(A-F) Measurement and quantification of TGF-α, IL-12/ IL-23, and IL-1β through flow cytometry analysis of mouse bone marrow-derived DCs treated with unloaded MSN, free TheraVac, MSN@ TheraVac, and LPS. *p < 0.05, **p < 0.01, ***p < 0.001, ****p < 0.0001, n.s. nonsignificant. Reproduced with permission 170. Copyright 2023, American Chemical Society. (G) Schematic representation of the SDT chemotherapy. (H) Mouse tumor growth in distinct treatment groups during the 15-day observation (n = 6). (I) Mean body weight change of mice after different treatments (n = 6). (J) NIR imaging of tumor-bearing mice at 0.5, 1, 2, 4, 8, 12, 24, and 48 hours after intravenous injection of free IR783 solution and MTN@DTX-CD-IR783. (K) Levels of DTX in tumors 20 hours following injection of DTX and MTN@DTX-CD (n = 3). (L) Spleen-associated DTX levels measured 20 h post-administration of DTX and MTN@DTX-CD (n = 3). (M) WBC, RBC, PLT cell counts in tumor-bearing mice with distinct treatments for 15 d (n = 6). (N) Fluorescence images displaying FITC (green) in tumors following a 12 h injection of MTN@FITC-CD, with and without US radiation. Scale bar: 50 Μm. (O) Images of H&E staining from spleens of mice that received 15 days of treatment with DTX and MTN@DTX-CD (US) are shown. The data are expressed as mean ± standard deviation. Scale bar: 50 μM. Reproduced with permission 171. Copyright 2015, American Chemical Society.

The most common method for developing the DDS using ROS-responsive MSN involves the direct conjugation of drugs to the nanoparticle surfaces via ROS-sensitive linkers. Yang *et al.* 170 developed mesoporous silica nanoparticles (MSN@TheraVac) that responded to ROS for colon cancer therapy. The nanoparticles had αPD-L1 attached to their surface through diselenide bonds, and they incorporated high mobility group nucleosome-binding protein 1 (HMGN1) along with resiquimod/R848. αPD-L was rapidly released under oxidative conditions, thereby avoiding both immune suppression and the exhaustion of effector T cells. Simultaneously, HMGN1 and R848 worked together to enhance the maturation of potent tumor-infiltrating dendritic cells (DCs), which in turn stimulated antitumor immune responses. Free TheraVac could be immediately used for DC stimulation, the TheraVac encapsulated in MSN@TheraVac required prior release. This likely induced delayed DC stimulation, which in turn promoted the secretion of Tumor Necrosis Factor-α (TNF-α) and Interleukin-12/Interleukin-23 (Figure 16A-F). Through intravenous injection therapy, MSN@TheraVac achieved a 100% cure rate in BALB/c mice with colorectal tumors. Other cancer vaccines can also be delivered using this ROS-responsive MSN platform, thereby achieving selective and effective immunotherapy.

Gated DDS maintain the drugs in a stable encapsulation state under normal physiological conditions through gating groups. In recent years, numerous stimulus-responsive, controllable drug release carrier systems have been developed using inorganic nanoparticles, synthetic polymers, peptides, cyclodextrins, and DNA/RNA as mesoporous “gating switches” for micropores. Zhang *et al.* 172 investigated a nanodelivery system (T/D@RSMSN) for drug release that is self-accelerating and triggered by ROS. The gated β-cyclodextrin (β-CD) group was linked to MSN through ROS-cleavable thioketal. The PEG chain of conjugated adamantane further modified the surface of the carrier through host-guest interactions. The DOX and ROS products, α-TOS, were encapsulated in the carrier. The intensity of fluorescence from DOX in MCF-7 cells showed that, under the action of α-TOS, the release rate of DOX (36 h) increased by 1.9 times. This indicated that the thioketal linker dissociated in the MCF-7 cells, after which DOX and α-TOS were released. The liberated α-TOS additionally elevated the levels of intracellular ROS and promoted the release of DOX. Similar to MSNs, MTNs have an appropriate drug-loading pore size and low cytotoxicity, making them another ideal carrier for stimulating reactive DDS. MTN is capable of producing substantial amounts of ROS by absorbing UV radiation or by focused US treatment. Zhang *et al.* 171 utilized the thioketal linker to connect β-CD to the outer surface of the MTNs as the gating group. This system encapsulated DTX. After two 40-second US treatments, the total release rate of DTX after 24 h was 96.8%. The release rate in the untreated group was only 7.5%. In a mouse model with tumors, focused US at the tumor location enhanced the efficacy of combination therapy and inhibited tumor growth. The tumors in the MTN@DTX-CD+US group decreased in size by approximately 60% within 15 days (Figure 16G-O).

## 6. Safety Assessment

The safety of ROS-responsive prodrug metabolites is crucial for their clinical translation. Although high concentrations of phenol have potential hepatotoxicity 173, the toxicity of boronate or boronic acid prodrug systems is relatively low. For instance, prodrug **2** can shrink tumors in mice without causing significant damage to the major organs. Sulfonic acid, a metabolite of thione bonds, is highly hydrophilic and is easy to clear 73. The prodrug **24** constructed based on this method, causes no significant damage to the major organs. In contrast, sulfide metabolites have higher polarity, are excreted more quickly, cause no apparent damage to organs, and have normal serum liver and kidney function indicators. Relatively few studies have been conducted on the safety of diselenide and selenium metabolites. However, the cytotoxicity of organic selenium is significantly lower than that of inorganic selenium 174. The drug carriers constructed based on this have good biological safety, with no noticeable damage to mouse organs detected in in *vivo* antitumor experiments. Furthermore, many studies cited in this article have achieved therapeutic effects through the augmentation of ROS levels, which seems to contradict the view that “high levels of ROS may promote tumor metastasis”. In fact, ROS exert a dual function in the occurrence and progression of tumors; therefore, there is no essential conflict between them and the treatment strategies based on ROS. Specifically, in the initial phases of cancer development, ROS levels that exceed the normal physiological range promote the formation and advancement of tumors 175. The primary mechanism involves the triggering of epithelial-mesenchymal transition (EMT) in tumor cells by activating signaling pathways like NF-κB and transforming growth factor-β (TGF-β), thereby enhancing the invasion and metastatic abilities of tumor cells 24. However, when ROS accumulate in large quantities within cells, their biological effects shift to anticancer effects. On the one hand, obstructing signaling pathways linked to proliferation, including the EGF/EGFR and PI3K/Akt pathways 176, impedes cell cycle progression, and such interference involves the arrest of the G2/M phase. It simultaneously reduces the synthesis of nucleotides and ATP, thereby suppressing the proliferative activity of cancer cells. Conversely, activation of the endoplasmic reticulum stress pathway 177, the mitochondrial apoptotic pathway 178, the p53-dependent apoptotic pathway, and the ferroptosis pathway 179,180, induces the demise of cancer cells. The multiple studies presented in this article have demonstrated the abilities of tumor cells to proliferate and migrate after drug treatment. For instance, prodrug **5** had a notable inhibitory impact on the proliferation and migration of MDA-MB-231 and MCF-10A cells and also decreased intracellular ATP content. Overall, the currently constructed ROS-responsive prodrug systems have metabolites with high hydrophilicity, rapid excretion, and low cytotoxicity in normal cells. In animal experiments, they have exhibited low organ toxicity. Moreover, ROS levels induced by treatment do not promote tumor metastasis. This provides strong experimental evidence and theoretical support for the clinical transformation of this type of prodrug system in terms of safety.

## 7. Conclusion

In conclusion, ROS-responsive DDS enable precise drug release and efficient delivery by exploiting elevated ROS levels in the TME. This presents an innovative approach to developing cancer treatments. This review systematically covers ROS-responsive platforms, from prodrug design and nanocarriers (such as polymers and inorganic nanoparticles) to intelligent hydrogels, highlighting their core design principles and recent advances. Response units, such as thioketal bonds, diselenide bonds, and phenylboronate esters, facilitate the breaking of chemical bonds or the conversion between hydrophilic and hydrophobic states. Tumor accumulation is enhanced when combined with the EPR effect or targeted modifications. Ultimately, controlled drug release occurs via ROS-triggered cascade reactions. These systems offer significant benefits, including reduced toxicity and side effects compared to traditional chemotherapy, enhanced effectiveness of immunotherapy, and prevention of tumor recurrence. Nonetheless, several key challenges remain: individual differences (tumor heterogeneity and varying ROS levels among patients) necessitate the development of intelligent systems capable of regulating response thresholds and addressing low ROS levels; integrating ROS-enhancing strategies, such as chemodynamic and photodynamic therapy, is essential to improve activation efficiency; adapting to the complex microenvironment requires multiple responsive mechanisms, including pH, enzymes, and GSH, as a single ROS response is insufficient for the dynamic TME; and issues related to the long-term biocompatibility of carrier materials, large-scale manufacturing, and in vivo metabolism warrant further study. Future research should focus on multidisciplinary approaches, employing artificial intelligence to predict personalized drug regimens and biomaterial science to optimize carrier design. The development of integrated diagnostic and therapeutic platforms that incorporate real-time imaging is vital. For clinical translation, establishing standardized evaluation systems is necessary to support the transition from animal experiments to human trials. As nanotechnology, immunology, and precision medicine continue to converge, ROS-responsive delivery systems are poised to overcome current barriers, thereby establishing a new standard for treating tumors and other diseases associated with oxidative stress.

## Figures and Tables

**Table 1:** Characteristics and reaction parameters of ROS-responsive groups.

ROS-responsive site	Group	Drug release process	Reaction specificity	Response concentration	Bond energy/Activation enthalpy	Refers
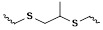	thioether	oxidation, hydrolysis	H_2_O_2_	50 mmol/L	272 kJ/mol	73,74,77-79
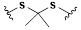	thioketal				240 kJ/mol	
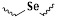	diselenide	oxidation, hydrolysis	H_2_O_2_	0.01% H_2_O_2_	244 kJ/mol	80,81
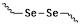	selenium ether				172 kJ/mol	
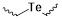	tellurium ether	oxidation	H_2_O_2_	100 μmol/L	228 kJ/mol	82-84
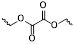	oxalate ester	nucleophilic substitution, hydrolysis	H_2_O_2_	10 μmol/L	ΔH^≠^≈46.9 kJ/mol	85,86
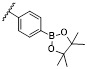	phenylboronate	Baeyer-Villiger oxidation-like rearrangement, hydrolysis, 1,6-elimination	H_2_O_2_	50 μmol/L	ΔH^≠^ ≈90-100 kJ/mol	87
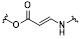	aminoacrylate	2+2 cycloaddition reaction	¹O₂	Producing ^1^O_2_ by photosensitizer	ΔH^≠^≈77-97 kJ/mol	76

**Table 2:** ROS-activatable prodrugs

Name	Drug	Activable group	*In vitro*/*in vivo* model	Therapeutic effect	Refs
1	DOX	Phenylboronate	4T1 H9C2	It retained the toxicity of DOX while reducing cardiotoxicity.	98
2 3 4	DOX	Phenylboronate	U87 MCF-7 HepG 2 MiaPaCa-2 A549	It had the highest activity in MiaPaCa-2 cells, with a prodrug conversion rate of approximately 40%.	99
5	Amonafide	Phenylboronic acid	MDA-MA-231 MCF-10A	It selectively inhibited DNA synthesis in tumor cells.	100
6 7	Crizotinib	Phenylboronic acid	H1993 H2228 RUMH	The prodrug exhibited the highest activity in H1993 cells, which had the highest ROS content.	102
8	Etoposide	Phenylboronate	HCT-116 xenografts in the BALB/c nude mice model	The tumor growth inhibition rate for the group administered a dose of 10mg/kg reached 46.19%.	103
9	β-Lap	Phenylboronic acid	Mia PaCa-2 (NQO1+) induced BALB/c mouse model	The tumor inhibition rates at doses of 20, 40, and 100 mg/kg were 54.27%, 67.52% and 71.64%, respectively.	104
10 11	FK886	Phenylboronic acid	293T Molt 4 PC-3	The ROS-sensitive FK866 prodrug was synthesized, which improved the targeting and efficacy of cancer treatment.	105
12 13	NAAF	Phenylboronate	BL-2 A2780 DU-145 Jurkat cells HDF	Prodrug 12 exhibited higher selectivity for cancer cells and had a lesser impact on normal cells.	106
14 15	4-FcAn	Phenylboronate	A2780	The IC_50_ values of prodrug **14** and prodrug **15** for A2780 cells were 13.8±1.0 and 8.3±0.9 μM, respectively.	107
16	GPX4 inhibitors	Phenylboronate	HT1080 OS-RC-2	Prodrug **16** showed stronger ferroptosis selectivity compared with GPX4 inhibitors.	108
17	HCPT	Thioketal bond	4T1 subcutaneous tumor-bearing mice model	The prodrug treatment group demonstrated the most significant anti-tumor effect and had relatively good safety.	111
18	CDOO-Me/SAHA	Thiolatic acid	A549 tumor xenograft BALB/c mice	The therapeutic effect of prodrug **18** was superior to that of the combined treatment group of CDOO-Me and SAHA.	109
19	F-OH-Evo	Thioether	U87 HeLa MCF-7 A549	Prodrug 19 exhibited an anti-tumor activity that was five times greater against U87 cells characterized by elevated integrin expression when compared to F-OH-Evo.	112
20	5-Fc	Phenylboronate	—	—	113
21	Fenretinide	Phenylboronate	HaCa T cells A431 SCaBER	After CPP treatment, the toxicity of prodrug **21** to different cell lines increased.	114
22 23	Pyrazolopyrimidinone	—	U-251MG	After being used in combination with CAP, the cytotoxicity of prodrug **22** and **23** increased by 15 times and 5 times, respectively.	115
24	CPT	Thioketal	A549	The apoptosis rate induced by prodrug **24** was as high as 99.6%.	116
25	Paclitaxel	Aminoacrylate	SKOV-3	When activated by light, the semi-inhibitory concentration values of prodrug **25** were 3.9 nM, respectively.	117
26	MMAE	3,5-Dihydroxybenzyl carbamate	4T1	The cell survival rate in the 4Gy+10 nM DHBC-MMAE group decreased to less than 30%.	110

**Table 3:** ROS-activatable polymeric nanoprodrugs.

Name	Drugs	Activable groups	*In vitro*/*in vivo* model	Therapeutic effect	Refs
DTX/PEG-PPMT	DTX	Thioether	A549 tumor xenograft BALB/c mice	Free DTX, DTX-loaded PEG-PPMT-11% PDL, and DTX-loaded PEG-PPMT-28% PDL nanoparticles inhibited tumor growth by 39%, 95%, and 93%, respectively.	121
MS-NP/Ce6&PTX	PTX	Thioether	MDA-MB-231	Combined phototherapy yielded the highest anti-cancer efficacy for MS-NP/Ce6&PTX.	122
PDA-Dox-Pc-QRH	DOX	Thioketal	A431 tumour-bearing mice	Laser-irradiated PDA-Dox-Pc-QRH eliminated tumors in approximately 10 days.	123
PF@PTX/Pt	PTX/Pt	Selenide	MDA-MB-231 HBL-100	PF@PTX/Pt showed concentration-dependent cytotoxicity against MDA-MB-231 and was not effectively activated in HBL-100 cells.	124
2BOH-TOS/DOX	DOX	boronate	MCF-7 MCF-7/ADR spheroids	The tumor growth rates observed in MCF-7 spheroids for Free DOX, 2BOH-TOS/NP, and 2BOH-TOS/DOX were -0.26, -0.31, and -0.51, while in MCF7/ADR spheroids, the rates were 1.84, 0.24, and -0.19, respectively.	125
mPEG-ROS-DOX	DOX	Thioketal	Balb/c mice bearing subcutaneous HepG2 tumors	Compared with mPEG-ROS-DOX, DOX had a more effective inhibitory effect on tumor growth; however, weight loss occured, with a maximum reduction rate of up to 20%.	126
Lapa@NPs	CPT	Thioketal	4T1 tumor-bearing mice with the tumor	On the 21st day, the tumor volume in the PBS treatment group rose significantly to 12.1 times, whereas the increase in tumor volume for the Lapa@NPs group was only 3.3 times.	129
TA-CA-Prodrug	PTX	Thioketal	4T1 tumor-bearing mice	The inhibition rates of tumor growth for the PTX group and the TA-CA-Prodrug group were 21.9% and 58.9%, respectively.	130
PTCD@B	DOX	Thioacetal	L929 4T1	PTCD@B has low toxicity to normal cells but shows significant cytotoxicity to 4T1 cells.	131
PEG-TK-DOX/PhA	DOX	Thioketal	CT 26 tumor-bearing mice	The group subjected to laser irradiation demonstrated a notable reduction in tumor size when contrasted with the non-irradiated group and the group receiving PBS injections.	132
Ce6@PPE-TK-DOX	DOX	Thioketal	Mice bearing MDA-MB-231 tumors	The Ce6@PPE-TK-DOX NPs(L+) group exhibited the highest anticancer efficacy.	133
PSPC NAs	CTX	Thioketal	Balb/c nude mouse model of A375 human melanoma	It had the potential to greatly reduce tumor growth while exhibiting low systemic toxicity and a high level of safety. The maximum tolerable dosage was notably greater than that of unbound medications.	134
DPPa NPs	CL	Thioketal	4T1 tumor-bearing mice	The tumor suppression rate of the DPPa NPs+ laser group was 85.8%.	135
ICG-PBT@NMPs	TPZ	Phenylboronate	4T1 tumor-bearing mice	ICG-PBT@NMPs had successfully achieved tumor inhibition through continuous bioadhesion.	136
RP-NPs	GEM	Thioketal	HeLa tumor-bearing nude mice	The weight of the tumor in the RP-NPs+US group was roughly one-third of that observed in the control group, demonstrating a therapeutic effect that outperformed that of RP-NPs used on their own.	137
ZnPc@Cur-S-OA	Cur	Thioketal	BALB/c mice inoculated with B16F10 cells	The ZnPc@Cur-S-OA + laser group showed significant tumor volume and load suppression effects after treatment.	138
Pro-5-FU@cLANCs	Pro-5-FU	Phenylboronate	A549 xenograft tumor model	Pro-5-FU@cLANCs achieved a tumor suppression rate of 73.1% and a survival rate of 80%.	144
^SS^CB_EPB+K_	DOX	Phenylboronate	orthotopic BC xenograft models in female C57BL/ 6 J mice	^SS^CB_EPB+K_ exhibited a similar performance to free EP in inhibiting tumor growth, with an extended survival time of over 60 days.	145
PHI@B/L	Lap/BDOX	Hydrazone bond/Phenylboronate	4T1 tumor-bearing mice	In comparison to the free DOX group, the PPHI@B/L group exhibited both the lowest tumor weight and volume, as well as a notable enhancement in tumor suppression and survival rates.	146
HCAG	GEM	Thioacetal	4T1 tumor-bearing mouse model	HCAG exhibited excellent in vivo anti-tumor effects at a dose of 10 mg/kg, with extremely low systemic toxicity.	147
ProCPT@P3	CPT	Phenylboronate	4T1 tumor model	Compared to CPT, ProCPT@P3 exhibited an enhanced inhibitory effect on tumor growth and a significantly reduced proliferation rate of tumor cells.	148
P-NM-Lapa	NM/Lapa	Phenylboronate	xenograft mouse models of HeLa cells	The tumor volume in mice treated with the P-NM-Lapa combined treatment was significantly reduced.	149
PDOX	DOX	Diselenide	HepG2 L02	PDOX demonstrated a toxicity that varies with dosage in HepG2 cells, resulting in a cell survival rate of 51%. Conversely, it displayed favorable cytocompatibility with LO2 cells.	150
PSD-Fc	PTX/DHA	Thioether	4T1 tumor-bearing mice	The mean tumor volume on day 22 was 1536 mm^3^ and 562 mm^3^ for mice administered with PBS and PSD-Fc, respectively.	156
^TK^NP_DHA_-Fc	PTX/DHA	Thioketal	4T1 tumor-bearing BALB/c mice	The average tumor mass of the ^TK^NP_DHA_-Fc group was 0.16±0.03 grams, which was 0.61 times and 0.42 times lower than that of the control groups NP_DHA_ and ^TK^NP_DHA_, respectively.	155
SN38-CA@FC	SN38	Thioacetal linker	xenograft LLC-bearing C57BL/6 mouse model	In the control group, the tumor volume rose to approximately 1250 mm³, while the tumor volume for SN38-CA@FC fell to 110 mm³.	157

**Table 4:** ROS-triggered hydrogel prodrugs.

Name	Drug	Activable group	*In vitro*/*in vivo* model	Therapeutic effect	Refs
P[(EG_3_SA)_x_-*co*-HEA_y_]	5-FU	Thioether	B16F10	The viability of B16F10 cells decreased by approximately 60% at 2 hours and dropped to approximately 80% after 48 hours.	161
hiROSponse^dox/ptx^	DOX/PTX	FeCp_2_	B16F10 melanoma-bearing mice	Compared with local injection of DOX without the use of a hydrogel matrix, this hydrogel could significantly reduce tumor growth by approximately 50%.	162
Dox/R848/aPD-1@Gel	DOX/R848/αPD-1	Methylthio group	B16F10 melanoma-bearing mice	The Dox/R848/aPD-1@Gel group demonstrated significantly stronger anti-tumor effects, with an average tumor volume of less than 300 mm^3^.	163
CMC hydrogels	DOX	Thioketal	HeLa	The cell survival rate of the DOX (20 μg) treatment group was approximately 6.5%, while that of the DOX+ICG encapsulated hydrogel (with equivalent DOX concentration) treated with near-infrared irradiation reached 6.7%.	164
TCO-PTX	PTX	-	MCF-7 cell spheroids	The prodrug activation process led to an increase in the number of dead cells within the cell spheres.	165
APPF hydrogels	αPD-L1	Thioketal	subcutaneous bilateral 4T1 tumor models	In the APPF + laser group, the average number of pulmonary metastatic nodules was found to be 0.8, indicating a reduction of 2.1 times compared to the APPF treatment group, and 16.7 times less than that observed in the PBS control group.	60

**Table 5:** ROS-triggered inorganic nanoprodrugs.

Name	Drug	Activable group	*In vitro*/*in vivo* model	Achievement	Refs
MSN@TheraVac	*α*PD-L1	Diselenide	Balb/c mice bearing an ectopic CT26 tumor model	After MSN@TheraVac treatment, the tumor-bearing mice initially exhibited slow tumor growth. Still, they then began to experience tumor shrinkage after the third treatment, and complete regression was finally observed after the fifth treatment.	170
T/D@RSMSNs	DOX/α-TOS	Thioketal	MCF-7 tumor bearing mice	The group that received injections of T/D@RSMSNs exhibited a notable reduction in tumor growth, and no systemic side effects were observed.	172
MTN@DTX-CD	DTX	Thioketal	S180 tumor bearing mice	In the MTN@DTX-CD+US group, the tumor volume decreased by approximately 60% within 15 days.	171

**Table 6:** Comparison of different ROS-responsive drug delivery systems.

System type	Core carrier/structure	ROS response mechanism	Targeting ability and delivery efficiency	Degree of functional integration	Safety
Small Molecule Prodrugs	Direct modification of drug molecules.	The drug is released through bond breakage.	Passive targeting (high ROS dependent on tumors), strong tissue penetration, but weak targeting.	The main focus is on drug release.	The structure is clear and the metabolic pathway is distinct.
Polymeric Nanoprodrugs	Polymer skeletons (such as PEG derivatives, polyamino acids).	Bond breakage or hydrophilic and hydrophobic transformation.	Active targeting (modified ligands) + passive targeting (EPR effect), with high tumor accumulation efficiency (3 to 5 times that of free drugs).	It can carry chemotherapy drugs, photosensitizers, etc., to achieve synergistic treatment.	Rely on the biocompatibility of polymers.
Hydrogel Prodrugs	Three-dimensional network hydrogels.	Key breakage or network expansion.	Local targeting (in situ injection retention) results in a long tumor retention time (several weeks), but the tissue penetration is limited.	It can integrate immune drugs to achieve synergistic treatment.	Long-term biocompatibility needs to be evaluated.
Inorganic Nanoprodrugs	Inorganic nanoparticles such as mesoporous silicon and titanium dioxide.	Modify the orifice gate, and ROS triggers release.	Passive targeting (EPR effect) can enhance targeting through surface modification and has a high drug loading capacity.	It can be integrated with imaging functions, such as MRI contrast and fluorescence labeling.	The long-term safety of inorganic degradation products needs to be verified.
